# Effect of PEGDMA Molecular Weight on the Physicochemical Properties of Thermoresponsive *N*-Vinylcaprolactam-Based Polymers

**DOI:** 10.3390/polym18212633

**Published:** 2026-10-23

**Authors:** Agnieszka Gola, Kinga Sadowy, Witold Musiał

**Affiliations:** Department of Physical Chemistry and Biophysics, Pharmaceutical Faculty, Wroclaw Medical University, Borowska 211, 50-556 Wroclaw, Poland; agnieszka.gola@umw.edu.pl (A.G.); kinga.gruszka@poczta.onet.pl (K.S.)

**Keywords:** functional polymers, medical polymers, smart polymers, polymer chemistry, *N*-vinylcaprolactam

## Abstract

Thermosensitive polymers P1–P5 of *N*-vinylcaprolactam (NVCL) and poly(ethylene glycol) dimethacrylates (PEGDMAs), av. Mn 750–20,000, were synthesized via surfactant-free precipitation polymerization (SFPP) using the cationic initiator 2,2′-azobis[2-methylpropionamidine] dihydrochloride (AMPA) at 70 °C. The polymerization course was monitored by conductivity. The physicochemical properties and thermoresponsive behavior of the polymers were investigated over the temperature range of 18–45 °C using dynamic light scattering, electrophoretic mobility measurements, surface tension, osmolality, attenuated total reflectance–Fourier transform infrared spectroscopy, and transmission electron microscopy. At 18 °C, all polymers formed nanoscale dispersed structures, with hydrodynamic diameters of approximately 23–41 nm. Heating induced pronounced changes in hydrodynamic diameter, with the magnitude of the response and transition temperature varying with PEGDMA chain length. The polymers differed in the extent of temperature-induced aggregation and in thermal hysteresis, reflecting differences in the reversibility of their temperature-induced changes in hydrodynamic diameter. Zeta potential and surface tension measurements revealed variations in interfacial properties among the polymer systems, whereas osmolality remained low and comparable between samples. Overall, variations in PEGDMA molecular weight were associated with differences in the physicochemical and thermoresponsive behavior of the NVCL-based polymer networks; however, no simple monotonic relationship between cross-linker molecular weight and the investigated properties was observed.

## 1. Introduction

The development of novel drug carriers that enhance the safety and efficacy of therapeutic agents represents a significant research challenge [1,2]. Targeted, controlled drug delivery systems enable delivery of active compounds to the intended location, reducing adverse effects and eliminating side-effects [3,4]. Controlled drug delivery systems also protect active ingredients from rapid degradation, increasing concentration in target tissues and reducing the required drug doses [5,6,7,8,9].

The size of the carrier is a pivotal parameter. Sizes ranging from a few tenths to a few hundred nanometers are favorable, as they promote carrier degradation and active substance release at the target site. Furthermore, the carriers must not be excessively small, as this could result in their capture by macrophages and subsequent blockage of blood vessels due to aggregation [10]. The utilization of nanocarriers has proven to be a valuable instrument in the formulation of numerous pharmaceutical compounds [11]. Tumor tissue has been observed to accumulate nanoparticles faster than other tissues due to leaky blood vessels. Without lymphatic drainage, this results in nanoparticle accumulation inside tumors. The concept has raised hopes of improving the therapeutic efficacy of chemotherapeutics [12,13,14,15,16,17].

Stimulus-responsive polymers, also known as ‘smart polymers’, are a class of compounds that exhibit a non-linear and reversible response to stimuli of endogenous or external origin, resulting in alterations to their physical and/or chemical properties. These compounds are characterized by their sensitivity to specific stimuli, including changes in enzymatic activity, pH, temperature, light, electric or magnetic fields, and chemicals. The development of these compounds is frequently motivated by the aspiration to emulate natural phenomena. The most prevalent applications involve changes in pH, temperature, redox potential, and light excitation [18,19,20]. In comparison with endogenous stimuli, exogenous stimuli offer a superior level of control and the capacity to induce faster drug release [21].

‘Smart’ polymers, which react reversibly to temperature stimuli, have been identified as some of the most promising carriers for controlled drug delivery [22,23,24]. The rationale behind this lies in the inherent property of these compounds to undergo phase transitions at a particular temperature [25]. It is noteworthy that temperature constitutes an essential physiological factor within the human body, with certain pathological conditions being characterized by temperature fluctuations [26]. A significant number of these compounds also undergoes phase transitions at temperatures close to the physiological temperature of the human body. Furthermore, these polymers can be modified to exhibit this change at the desired temperature [27,28,29,30].

A change in solubility is accompanied by a conformational change in the structure of the polymer. The temperature at which the polymer structure or solubility undergoes a phase transition is designated the transition temperature. Thermosensitive polymers can be categorized into two distinct groups: polymers characterized by an upper critical solution temperature (UCST) and polymers with a lower critical solution temperature (LCST) [24]. The behavior of LCST is attributable to the unique equilibrium between the hydrophilic and hydrophobic components of the monomer. It is evident that LCST is significantly influenced by the capacity of the polymer to establish hydrogen bonds with water. It can thus be concluded that the transition temperature can be increased by adding hydrophilic monomers to the polymer chain. This results in an increase in the number of hydrogen bonds, consequently shifting the phase transition temperature towards higher values. Conversely, the incorporation of hydrophobic monomers into the polymer chain results in a minimalization of hydrogen bonds and, consequently, a decrease in the LCST. Therefore, copolymerization with hydrophilic or hydrophobic monomers emerges as a prevalent and efficient strategy to modulate the LCST to a desired value [31,32,33,34,35].

In comparison to the extensively documented polymers that exhibit LCST, polymers with the opposite behavior, i.e., polymers that are dissolved above UCST in aqueous solution, have received comparatively less attention. This is primarily due to the greater difficulty in achieving this behavior in aqueous solutions, and secondly due to the fact that the UCST transition temperature is very sensitive to the environment. The LCST behavior of polymers in water is based on entropy-driven dehydration, while the UCST behavior in water requires strong supramolecular attraction of polymer chains [36,37,38,39].

Stimuli-responsive polymers can control drug release profiles and interactions with cells. Stimuli-responsive drug carriages undergo a change in their physicochemical properties in response to changes between normal and tumor tissues, or to physical stimulation, to increase drug uptake and release [40,41]. In addition, polymers that are characterized by an LCST have the advantage that they can be utilized in injectable formulations [42,43,44]. Such drug carriers can be injected into the tumor tissues, for example [45]. It will form within the available free volume and adapt to the surrounding tissue [46].

Poly *N*-vinylcaprolactam (PNVCL) is a thermosensitive polymer with an LCST of 32–50 °C. The LCST value of the PNVCL is contingent upon the polymer concentration, its molecular weight, and the composition of the solution [47,48]. The merits of PNVCL are attributable to its properties, which stem from its biocompatibility, thermosensitivity within the human body temperature range, and solubility in water. In addition to these properties, PNVCL is stable against hydrolysis and non-toxic. Research has demonstrated the capacity of PNVCL to inhibit certain strains of pathogenic bacteria, including *Escherichia coli* (Gram negative) [49], *Staphylococcus aureus* (Gram positive) [50] and *Candida albicans* yeast [51]. These properties render PNVCL a compelling candidate for biomedical applications [52].

The polymerization process demands application of a specific initiator which enables creation of active radicals. The polymeric chain grows on the initiated radical particle, and elongates, depending on the availability of the monomer. The smaller the amount of the available monomer, the shorter the macromolecule chain. In former studies [53] we applied relatively low concentrations of monomer, which led to nanoparticles, with possible aggregation patterns. Thus it was interesting to perform further experiments in the low-concentration range of the monomer, which could result in synthesis of alternatively thermosensitive particles, with aggregation patterns, during LCST acquisition with temperature increase. Also the dissociation of the initiator, as well as the double bond breakage may be altered at increased initiator concentrations. Some researchers used an increased initiator concentration, which resulted in relatively small macromolecules [54,55]. Machado et al. stated that the initiator dissociation may have high influence on the resulting size of the obtained molecules [56]. Xu et al. evaluated the process of butyl acrylate polymerization in anionic microemulsion, and observed the important influence of initiator on the length of the obtained polymeric chains [57].

Polymer crosslinking is defined as the process of creating macromolecules with a spatial, three-dimensional structure and can occur in two different stages of polymer synthesis: during the formation and growth of polymer chains, and after their synthesis [58,59].

Cross-linked colloidal particles, also known as microgels and nanogels, have been the focus of recent extensive research [60]. Various types of polymer-based microgels have been shown to exhibit smart behavior. In particular, microgels based on NIPA and NVCL have attracted significant attention [61,62,63].

Polyethylene glycol dimethacrylate (PEGDMA) serves as a crosslinking agent and is also employed as a useful raw material for chemical syntheses. Hydrogels synthesized from PEGDMA exhibit minimal toxicological response and can be modified to be biologically inert or biocompatible [64,65]. Cross-linked PEG-based polymers (hydrogels) are a suitable material for use in drug delivery systems [66,67].

The objective of this study was to investigate the influence of PEGDMA molecular weight (Mn 750–20,000) on the physicochemical properties and thermoresponsive behavior of NVCL-based polymer systems synthesized under otherwise identical nominal reaction conditions. The experimental characterization focused on temperature-dependent hydrodynamic diameter, dispersity, zeta potential, thermal hysteresis, and other physicochemical properties of the obtained polymer dispersions. The present findings contribute to the physicochemical assessment of NVCL-based polymer systems and provide a basis for further investigations into their potential pharmaceutical applications.

## 2. Materials and Methods

### 2.1. Materials

*N*-vinylcaprolactam (NVCL, 98%, St. Louis, MO, USA), 2,2′-Azobis(2-methylpropionamidine) dihydrochloride (AMPA, 97%, Sigma-Aldrich, Steinheim, Germany), and poly(ethylene glycol) dimethacrylate (PEGDMA) (average Mn ~750, ~2000, ~6000, ~10,000, ~20,000, St. Louis, MO, USA), were obtained from Sigma-Aldrich and used without further purification. The dialysis tubing cellulose membrane (MWCO 12,000–14,000 Da) was also purchased from Sigma-Aldrich (St. Louis, MO, USA). The deionized water (<0.06 μS cm^−1^) was filtered using an HLP 20 system (microfiltration capsule 0.22 μm, Hydrolab, Straszyn, Poland) and complied with the requirements of the PN-EN ISO 3696:1999 standard for analytical laboratories [68].

### 2.2. Synthesis

**Figure 1:**
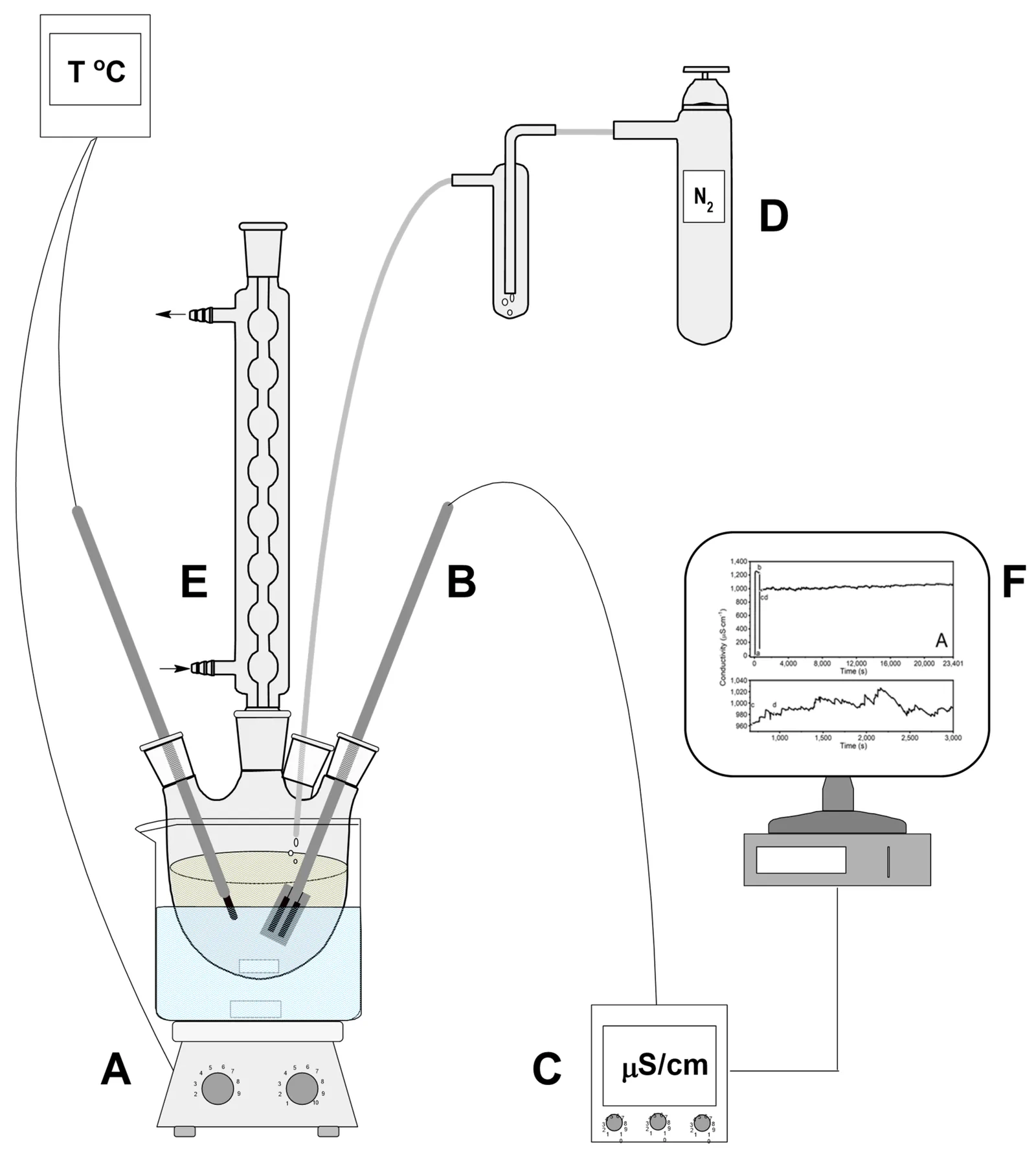
General diagram of the apparatus set including a magnetic stirrer (**A**) with thermostat function, a conductivity sensor (**B**) with a measuring set (**C**), a nitrogen inlet (**D**), a cooler (**E**), and a computer set (**F**) for recording conductivity data during synthesis. The arrows indicate the cooling water inlet and outlet.

The synthesis of five thermosensitive polymers (P1–P5) was accomplished through surfactant-free precipitation polymerization (SFPP). PEGDMA was utilized as the crosslinking agent and AMPA as the cationic initiator. The reaction was carried out for 6 h at 70 °C in an aqueous medium at a total volume of 1000 mL, under continuous stirring (2500 rpm) in an inert N_2_ atmosphere. In the present paper, an alternative method of monitoring the process of free radical polymerization, performed as SFPP, is proposed. This method involves the online, real-time measurement of the electrical conductivity of the reaction mixture. The observed phenomena during the process may be reflected by the changes in conductance within the mixture, thus providing information on the state of the system at specific time intervals. This work provides a synopsis of the preceding observation and was implemented in a particular system with cross-linkers that varied according to the carbon chain length. The proposed system applied in the process of the synthesis is presented on Figure 1.

The molar ratio of the reactants utilized in the reaction remained constant in all syntheses, i.e., NVCL:AMPA:PEGDMA = 1:0.25:0.0005. The list of substrates, products, and their nomenclature/acronyms can be found in Table 1.

The selection of substrate ratios in the reactions was based on the results of our previous studies [53], as well as on the assumption of an increased initiator content. The relatively high AMPA concentration was deliberately selected based on our previous experimental findings [53], which demonstrated a non-monotonic relationship between initiator concentration and hydrodynamic diameter, with approximately 1.46 g/L yielding the smallest stable PNVCL nanoparticles under the investigated synthesis conditions. The increased initiator concentration is primarily employed to synthesize low-molecular-weight polymers and to mitigate the loss of initiating radicals in undesirable reactions, such as those involving water as a solvent. It can also prevent excessively low local initiator concentrations, which can affect the efficiency of the polymerization process. An elevated initiator concentration was introduced to increase the number of active centers generated during polymerization, thereby accelerating chain propagation and producing polymer strands with a lower degree of polymerization. Such conditions are conducive to the formation of networks with a more homogeneous microstructure, while concomitantly suppressing the formation of microgels. This is advantageous when comparing systems that differ solely in the length of the PEG-based cross-linker. Furthermore, the utilization of a higher initiator concentration serves to amplify structural disparities emanating from variations in cross-linker length, a factor that is of particular importance when assessing thermoresponsive behavior and alterations in polymer coil dimensions. In the polymerization experiments, reduced concentrations of PEGDMA were employed in order to prevent excessive crosslinking and to avoid premature gelation of the reaction mixture. Lower amounts of crosslinking agents enable greater precision in terms of network density, thereby promoting the formation of polymer coils with a reduced number of junction points. This approach facilitates a clearer assessment of how the chain length of the cross-linker influences the physicochemical properties of the resulting polymers, particularly their responsiveness to temperature changes.

The experiments conducted provide compelling evidence that validates the approach and yielded noteworthy results that may serve as a foundation for future research.

The reaction mixture underwent purification through forced equilibrium dialysis (FED) using deionized water, followed by lyophilization for 26 h with an Alpha 1-2 LD freeze dryer (Martin Christ Freeze Dryers, Osterode am Harz, Germany). The obtained polymers were characterized using ATR-FTIR and TEM methods.

### 2.3. Conductivity, pH and Osmolality Measurements

The CC-505 conductometer (accuracy up to 19.999 mS·cm^−1^ ± 0.1%, from 20.000 mS·cm^−1^ ± 0.25%, Elmetron, Zabrze, Poland) was utilized to measure the conductivity of the reaction mixture, during polymerization at a constant temperature of 70 °C, and subsequently used for synthesis during the cooling process. The conductometer was equipped with an EC-60 immersion conductivity sensor with platinum electrodes, a glass case (K = 1.0 ± 0.2 cm^−1^, Elmetron, Zabrze, Poland) and temperature sensor Pt-1000A (0–100 ± 0.35 °C). The conductivity and temperature sensors were continuously immersed in the reaction mixture. Temperature compensation was ensured both during the polymerization reaction and the cooling process. Manual intervention was applied to the former, while automatic mechanisms were utilized in the latter.

The ELMETRON CPC-511 apparatus (range −2.00 ÷ 16.00 pH, accuracy ±0.01 pH, Elmetron, Zabrze, Poland) was utilized to measure pH. The polymers’ pH values were measured at ambient temperature before and after the purification process using an ELMETRON EPS-1 electrode (Elmetron, Zabrze, Poland).

Osmolality of purified polymer samples was determined using a Marcel OS 3000 microprocessor-based osmometer (Marcel, Warsaw, Poland), which utilizes the freezing point depression method. The device measured the decrease in the crystallization temperature of the supercooled sample with respect to the pure solvent, and the obtained values were automatically converted by a microprocessor and expressed in mOsm kg^−1^ H_2_O. Samples were prepared by dissolving the lyophilized samples in purified water at a concentration of approximately 0.015 g/10 mL. Each measurement was performed with 100 μL of polymer solution. Measurements were performed in five independent replicates for each sample.

### 2.4. Attenuated Total Reflection Fourier Transform Infrared Spectroscopy (ATR-FTIR) Measurements

The Nicolet iS50 FT-IR spectrometer, equipped with a diamond crystal sampling accessory (Thermo Fisher Scientific, Madison, WI, USA) and a deuterated L-alanine-doped triglycine sulfate detector (DLaTGS) was utilized to conduct ATR-FTIR. The prepared samples were analyzed in the wavenumber range 4000 to 400 cm^−1^ with an average of 32 scans at resolution 4 cm^−1^ ± 0.01 cm^−1^. The ATR module was cleaned with methanol and dried before each analysis. The background spectrum was obtained using a blank ATR crystal and automatically subtracted from the sample spectrum. The ATR-FTIR spectra of commercial substrates and the lyophilized products were measured under equal instrument conditions at ambient atmosphere and temperature. The processing of the spectral data was carried out using OMNIC software (version 9, Thermo Fisher Scientific, Madison, WI, USA), and qualitative analysis was performed to compare the substances.

### 2.5. Hydrodynamic Diameter (HD) and Polydispersity Index (PDI) Measurements

The Zetasizer Nano ZS ZEN3600 device (Malvern Instruments, Malvern, UK) with a red He-Ne laser (4 mW, λ = 633 nm) and scattering angle of 173° was utilized to assess the hydrodynamic diameter (HD), distributions, and polydispersity index (PDI) of aqueous polymer particle dispersion through a dynamic light scattering (DLS) technique. The Non-Invasive Back Scatter (NIBS) method was utilized. The light intensity during measurement was regulated by a laser beam attenuator. In total, 1 mL of each purified polymer dispersion (not diluted/precipitated after dialysis) was transferred to a translucent polyacrylic disposable cuvette DTS-0012, (Malvern Instruments, Malvern, UK) and placed in a temperature-controlled measurement cell. The sample was allowed to stabilize at the set temperature for 240 s. Measurements were taken in five independent repetitions. The number of runs per measurement was automatically adjusted between 10 and 100. DLS measurements were recorded in 1 °C intervals from 18 to 45 °C. The cumulants analysis algorithm estimated the HD and PDI parameters using the methodologies outlined in ISO documents: ISO 13321:1996E and ISO 22412:2008 [69,70,71]. The refractive index and viscosity of water were used as dispersant parameters and polystyrene latex was the material parameter. The size distribution was presented by intensity, with a PDI value of 0 for the highly monodispersed standard. Zetasizer^®^ software (version 7.10, Malvern Instruments, Malvern, UK) was used to create custom SOPs for samples and process DLS data. The statistical analysis was conducted using descriptive methods.

In order to assess the reversibility of the thermoresponsive properties of the synthesized polymers, thermal hysteresis measurements were carried out by monitoring the hydrodynamic diameter during heating (18–45 °C) followed by subsequent cooling (45–18 °C). An equilibration time of 300 s was applied at each measurement point. Measurements were recorded during the entire heating and cooling cycles, with a temperature step of 1 °C in the ranges of 18–30 °C and 36–45 °C, and an increased temperature resolution of 0.5 °C in the region of 30–36 °C, where the phase transition was observed. The phase transition temperature was determined by fitting the experimental data to the Boltzmann function, with the inflection point parameter (x_0_) taken as the characteristic phase transition temperature, corresponding to the temperature at which the steepest change in hydrodynamic diameter occurred. The fitting range was selected individually for each polymer sample and for each heating and cooling cycle. The selected temperature range represents the narrowest interval covering the phase transition region that enabled stable Boltzmann fitting and provided physically meaningful transition temperatures, while avoiding regions dominated by extensive aggregation and minimizing over-parameterization of the fitting model. Thermal hysteresis (ΔT) was calculated as the difference between the phase transition temperature obtained during cooling and that determined during heating. For one polymer sample (P2), due to the limited number of experimental points within the phase transition region, one fitting parameter was constrained to improve the stability of the fitting procedure. The Boltzmann fitting was performed using OriginPro 2026b (OriginLabCorporation, Northampton, MA, USA)

### 2.6. Surface Tension Measurements (ST)

The automatic laboratory tensiometer D-MT1A (POLON-IZOT Sp. z o.o., Warsaw, Poland) was utilized to determine the surface tension of the polymer dispersions using the Du Noüy ring method. Measurements were performed at four selected temperatures ranging from approximately 23 to 38.5 °C, covering the phase transition temperature region. Approximately 10 mL of each polymer dispersion was used for the measurements. The polymer samples were prepared at the same concentration as those used for osmolality measurements (approximately 0.015 g of lyophilized polymer dissolved in 10 mL of purified water). For each polymer and temperature, at least three measurements were performed on the same sample, with up to five measurements obtained when necessary. Before each temperature change and before measuring a new polymer sample, the measuring ring was cleaned by flaming to remove any residue from the previous measurement. Deionized water was measured under the same experimental conditions and used as a reference.

### 2.7. Refractive Index Measurements (RI)

The DR6100-TF digital refractometer (A.KRÜSS Optronic GmbH, Hamburg, Germany) was used to determine the refractive index of the polymer dispersions as a function of temperature. The instrument was equipped with a sapphire measuring prism and an integrated Peltier temperature control system. The measurements were conducted at a wavelength of 589 nm. The refractometer operates over a refractive index range of n*_D_* 1.3200–1.7000, with a resolution of 0.0001 and an accuracy of ±0.0001 n*_D_*. Refractive index values were recorded at 20, 25, 35, 38, 40, and 45 °C. The temperature was controlled with an accuracy of ±0.1 °C. Deionized water was measured at the same temperatures and used as a reference. Measurements were performed in triplicate and the results are expressed as mean ± SD. Polymer dispersions were prepared at a concentration of 1.5 mg/mL, using the same procedure as described for the surface tension and osmolality measurements.

### 2.8. Zeta Potential Measurements (ZP)

The Zetasizer Nano ZS ZEN3600 device (Malvern Instruments, Malvern, UK) was utilized to determine the zeta potential of polymer particles in aqueous dispersion using laser Doppler velocimetry (LDV). The Henry equation was approximated using the Smoluchowski model (f(Ka) = 1.5). The measurements were obtained using a 0.75 mL U-shaped plastic capillary cuvette with integrated gold-plated copper electrodes (Malvern Instruments, Malvern, UK). The temperature range was set from 18 to 45 °C, with measurements recorded at each temperature at 1 °C intervals and an equilibration time of 120 s. The ZP value was determined by averaging five measurements at each temperature. The measurements were recorded with Zetasizer^®^ software (version 7.10, Malvern Instruments, Malvern, UK), and the statistical analysis was conducted using descriptive statistical methods.

### 2.9. Transmission Electron Microscopy Measurements (TEM)

A FEI Tecnai G2 20 X-TWIN transmission electron microscope (FEI Company, Hillsboro, OR, USA) operated at 200 kV and was equipped with an electron gun with the LaB6 cathode, an FEI Eagle 2K CCD camera, and a HAADF detector for the STEM scanning; the transmission technique was utilized to characterize the morphologies of polymer particles. The specimens were prepared by transferring a drop (~30 µL) of polymer particles in aqueous dispersion onto a 400-mesh carbon-coated copper grid (3–4 nm). The samples were then left to dry for approximately 30 min in the air before measurements could be taken. The data were processed using TEM Imaging & Analysis software (version 3.2 SP6).

## 3. Results

### 3.1. Synthesis Measurements

**Figure 2:**
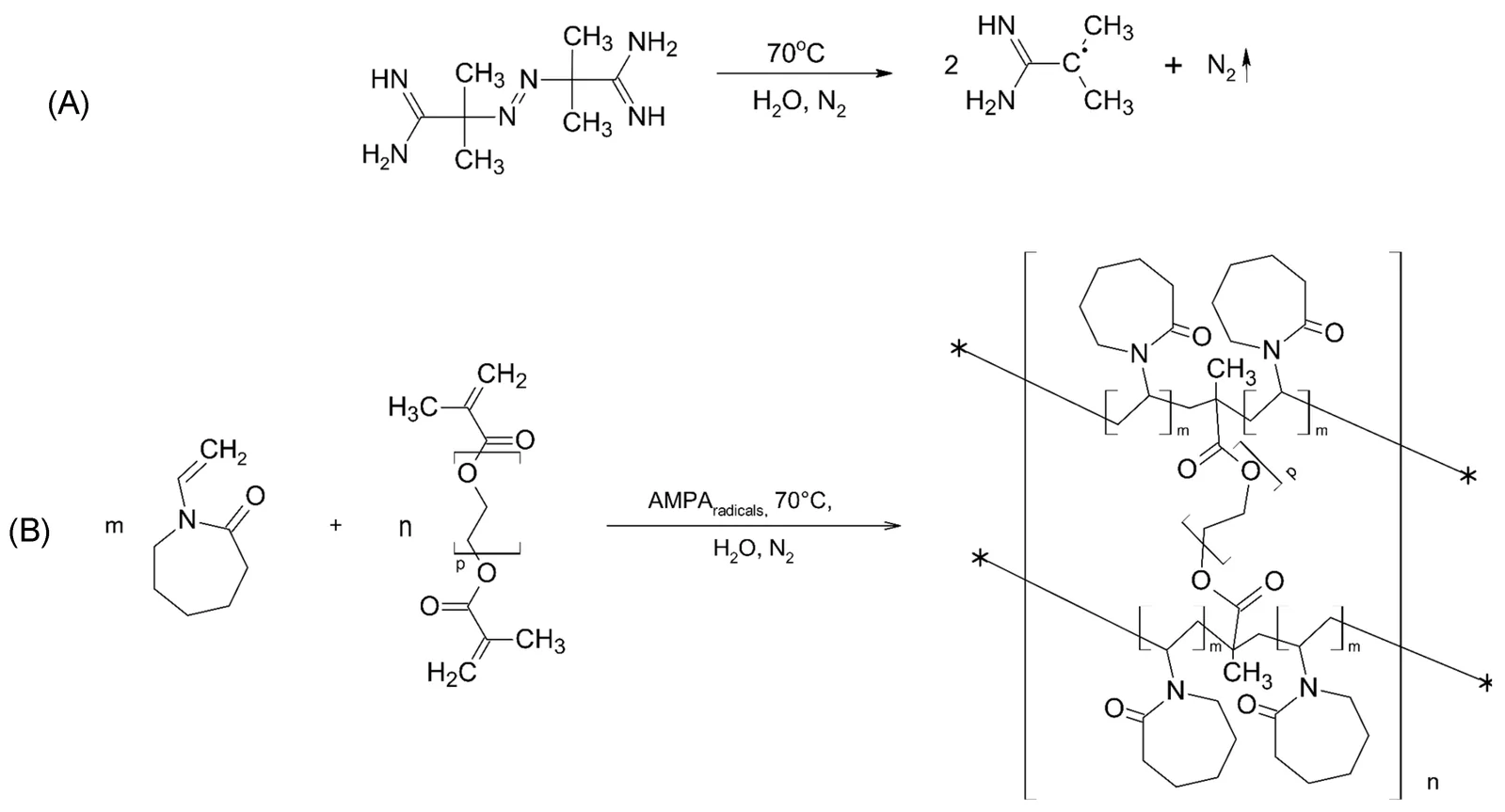
General scheme of the polymerization reaction of NVCL: (**A**) radical generation; (**B**) polymerization involving the use of a cross-linker (PEGDMA) under the experimental conditions employed in this study, together with the suggested polymer structure. Asterisks indicate continuation of the polymer chains.

As illustrated in Figure 2, a generic scheme of free radical polymerization is presented, in which five P1–P5 polymers were synthesized through the utilization of the SFPP technique.

Each synthesis was carried out under the same reaction conditions. However, the individual syntheses differed in terms of the use of a crosslinking agent with varying carbon chain lengths. Section 2.2 provides a more detailed description of the polymerization procedure and the amounts of substrates utilized (cf. Table 1).

The polymerization products—aqueous polymer dispersions, purified by forced equilibrium dialysis—were subjected to lyophilization. The resultant freeze-drying process yielded a solid wattle-like product.

From 100 mL of purified polymer solutions, the following quantities of P1, P2, P3, P4 and P5 polymers were obtained through freeze-drying: 0.1567 g, 0.1294 g, 0.1320 g, 0.1505 g, and 0.1355 g.

### 3.2. Conductivity, pH and Osmolality Measurements

**Figure 3:**
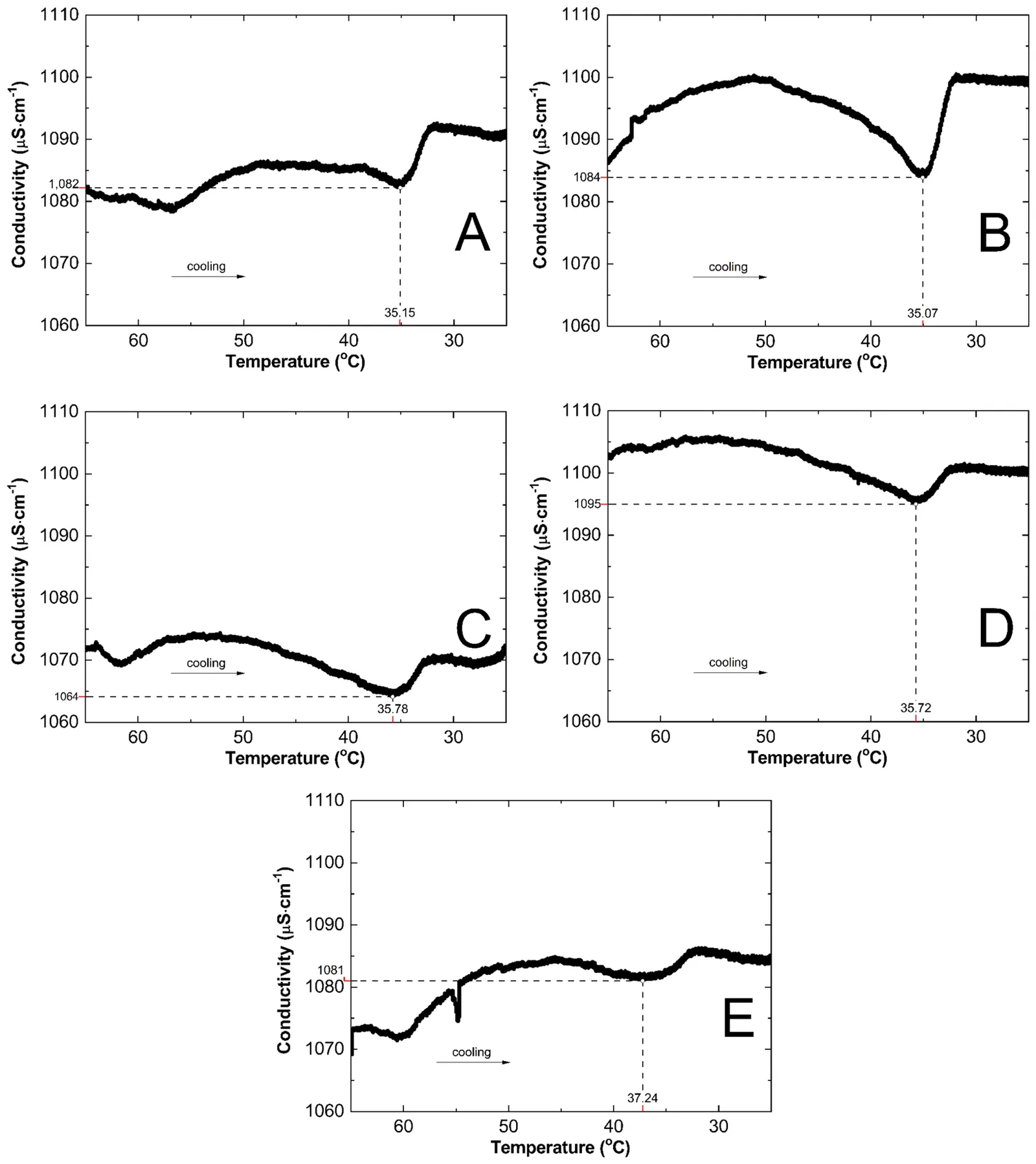
Alterations in conductivity in relation to temperature in the post-reaction mixtures of P1 (**A**), P2 (**B**), P3 (**C**), P4 (**D**) and P5 (**E**) during cooling.

The results of the conductivity measurements of the reaction mixtures P1–P5 as a function of temperature during cooling to room temperature are displayed in Figure 3A–E. The PEGDMA 20000 cross-linked polymer P5 showed the highest conductivity at minimum temperature during cooling (37.24 °C), with the minimum being broader and less pronounced compared to the other polymer systems. 

The pH values measured at room temperature for the unpurified polymer dispersions (P1–P5) were as follows: 5.22 (P1), 4.81 (P2), 5.00 (P3), 5.39 (P4) and 4.94 (P5). After purification by forced equilibrium dialysis, the pH values increased to 6.50 (P1), 6.35 (P2), 6.71 (P3), 6.52 (P4) and 6.71 (P5).

Furthermore, the osmolality of the purified polymer dispersions was determined. All investigated systems exhibited remarkably low osmolality values ranging from 2.0 to 2.6 mOsm kg^−1^ (with mean values of five independent measurements).

### 3.3. ATR-FTIR Measurements

**Figure 4:**
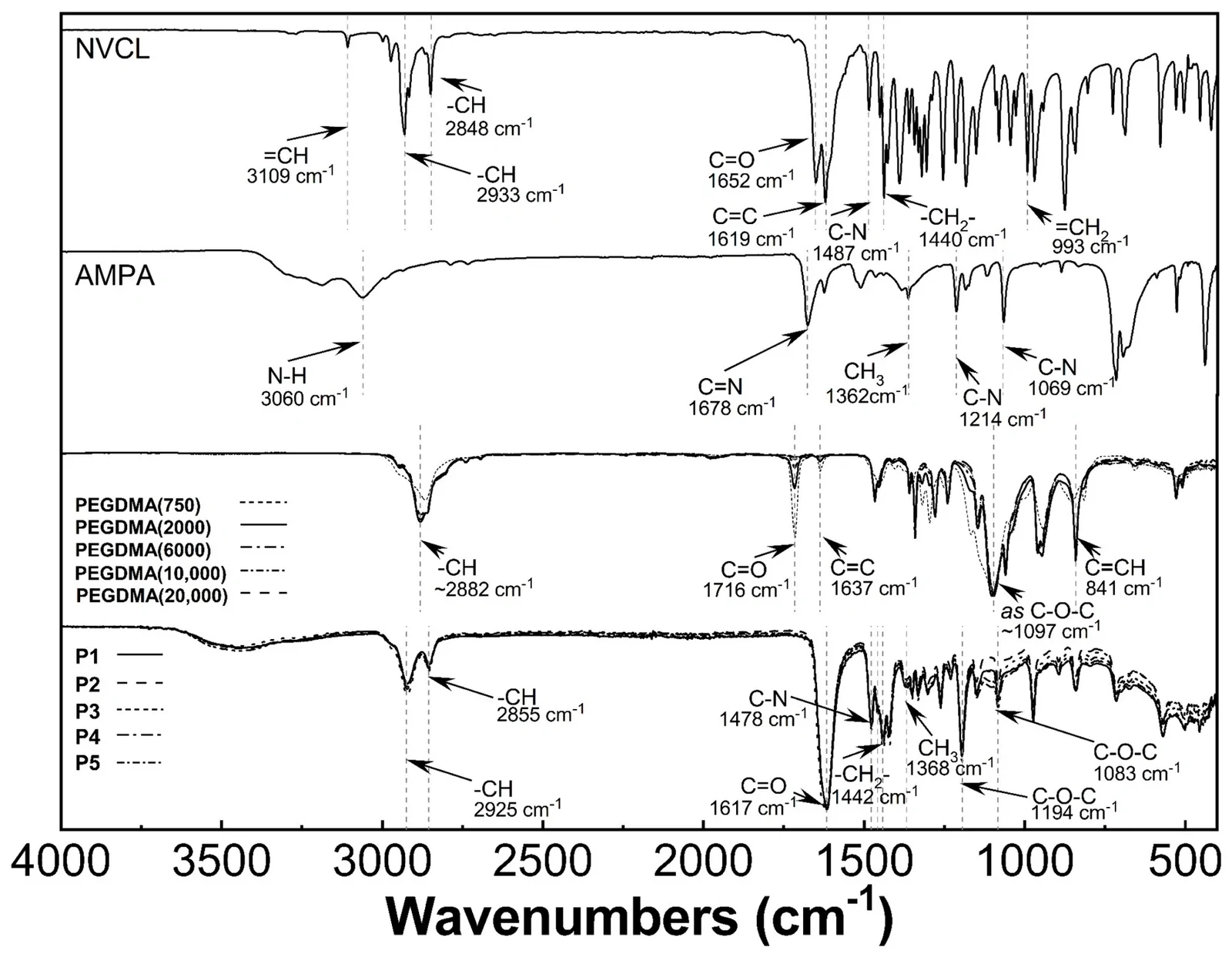
Fourier-transform infrared spectroscopy with attenuated total reflectance (ATR−FTIR): spectra of the monomer *N*-vinylcaprolactam (NVCL), initiator 2,2′-azobis[2-methylpropionamidine] dihydrochloride (AMPA), cross-linkers (PEGDMA), and synthesized polymers P1−P5.

The ATR-FTIR infrared spectroscopy spectra for the monomer NVCL, the initiator AMPA, the five cross-linker PEGDMAs and the synthesized polymers P1, P2, P3, P4 and P5 are shown in Figure 4. Peaks characteristic of the individual compounds are marked and described on the spectra. A comprehensive analysis of the NVCL, AMPA and PEGDMAs spectra can be found in our earlier publications [53,72].

### 3.4. HD and PDI Measurements

**Figure 5:**
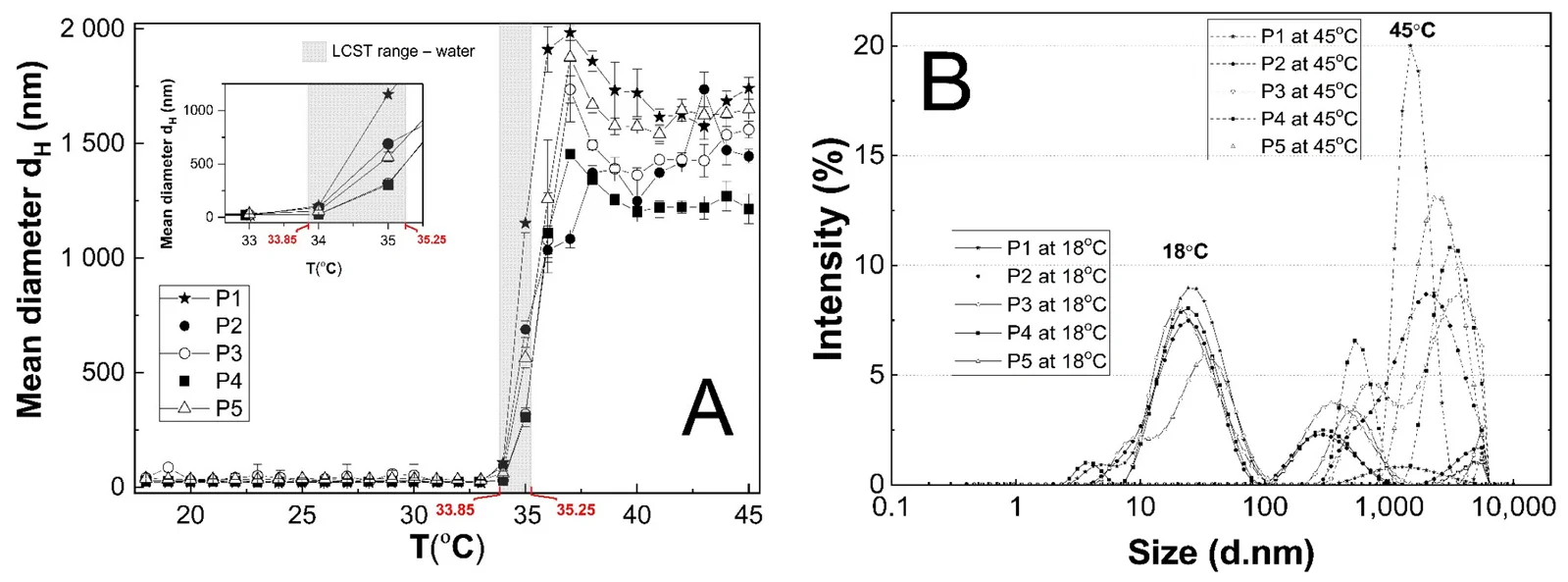
(**A**) Alterations in hydrodynamic diameter in relation to temperature of the P1, P2, P3, P4 and P5 samples, as determined by dynamic light scattering; (**B**) intensity-weighted hydrodynamic diameter distributions for P1, P2, P3, P4, and P5 dispersions, in five measurements, at 18 °C for the solid line and 45 °C for the dashed line, as determined by dynamic light scattering.

**Figure 6:**
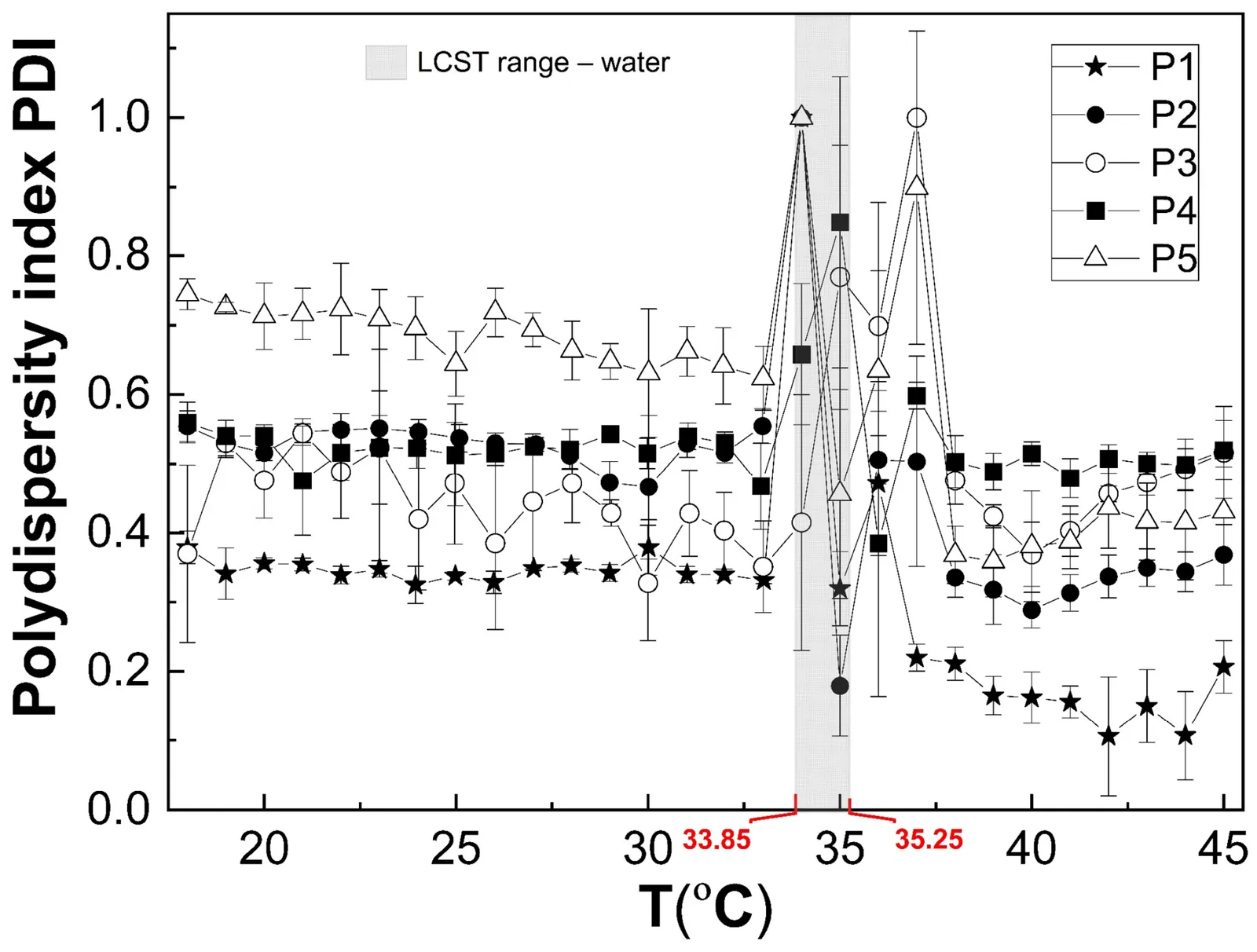
Alterations in the polydispersity index in relation to the temperatures of samples P1, P2, P3, P4 and P5, as determined by dynamic light scattering.

Figure 5A and Figure 6 illustrate the mean HD and PDI values, respectively, derived from five assessments of purified aqueous dispersions of polymers P1–P5, conducted at a single temperature within the temperature range of 18–45 °C.

The pronounced variations in HD and PDI values at particular temperatures suggest that these temperatures correspond to a phase transition. As is evident in the graphs, the HD and PDI variations indicative of LCST demonstrate a high degree of consistency between each other.

As illustrated in Figure 5B, the intensity-based distribution of cross-linked polymer particles (P1–P5) in an aqueous dispersion is presented at two different temperatures: 18 °C (solid line) and 45 °C (dashed line).

### 3.5. Thermal Hysteresis Measurements

**Figure 7:**
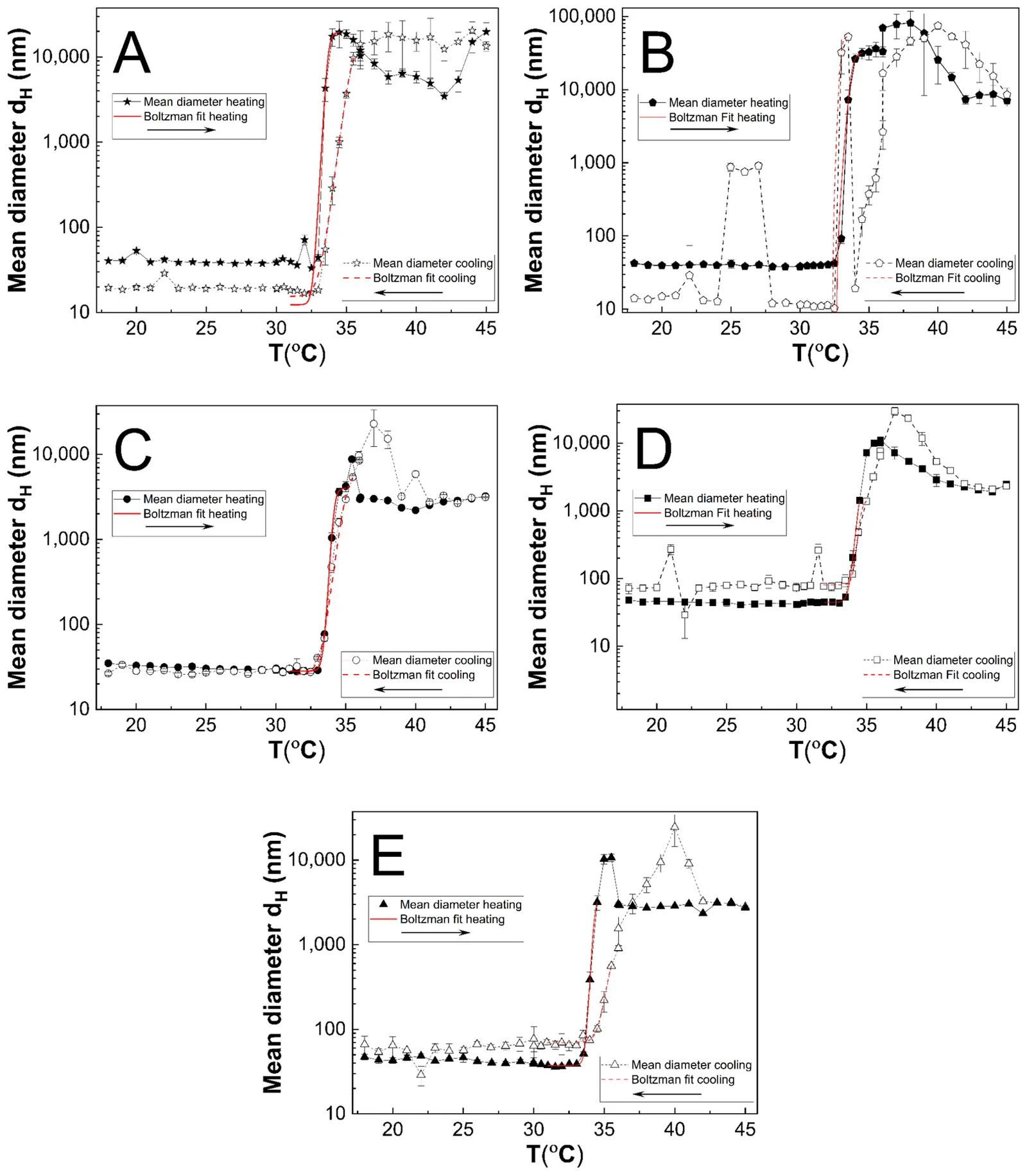
Thermal hysteresis profiles of hydrodynamic diameter as a function of temperature for polymers P1 (**A**), P2 (**B**), P3 (**C**), P4 (**D**), and P5 (**E**). Solid lines represent the corresponding Boltzmann fits.

The reversibility of the thermoresponsive behavior was further investigated by thermal hysteresis analysis during heating and cooling cycles. The hydrodynamic diameter profiles obtained from these measurements are shown in Figure 7, and the calculated transition temperatures and hysteresis values (ΔT) are listed in Table 2.

Except for polymer P2, all Boltzmann fits were performed without parameter constraints. For the cooling cycle of polymer P2, one fitting parameter was fixed because of the limited number of experimental points within the selected phase transition region, thereby ensuring stable Boltzmann fitting.

### 3.6. St Measurements

**Figure 8:**
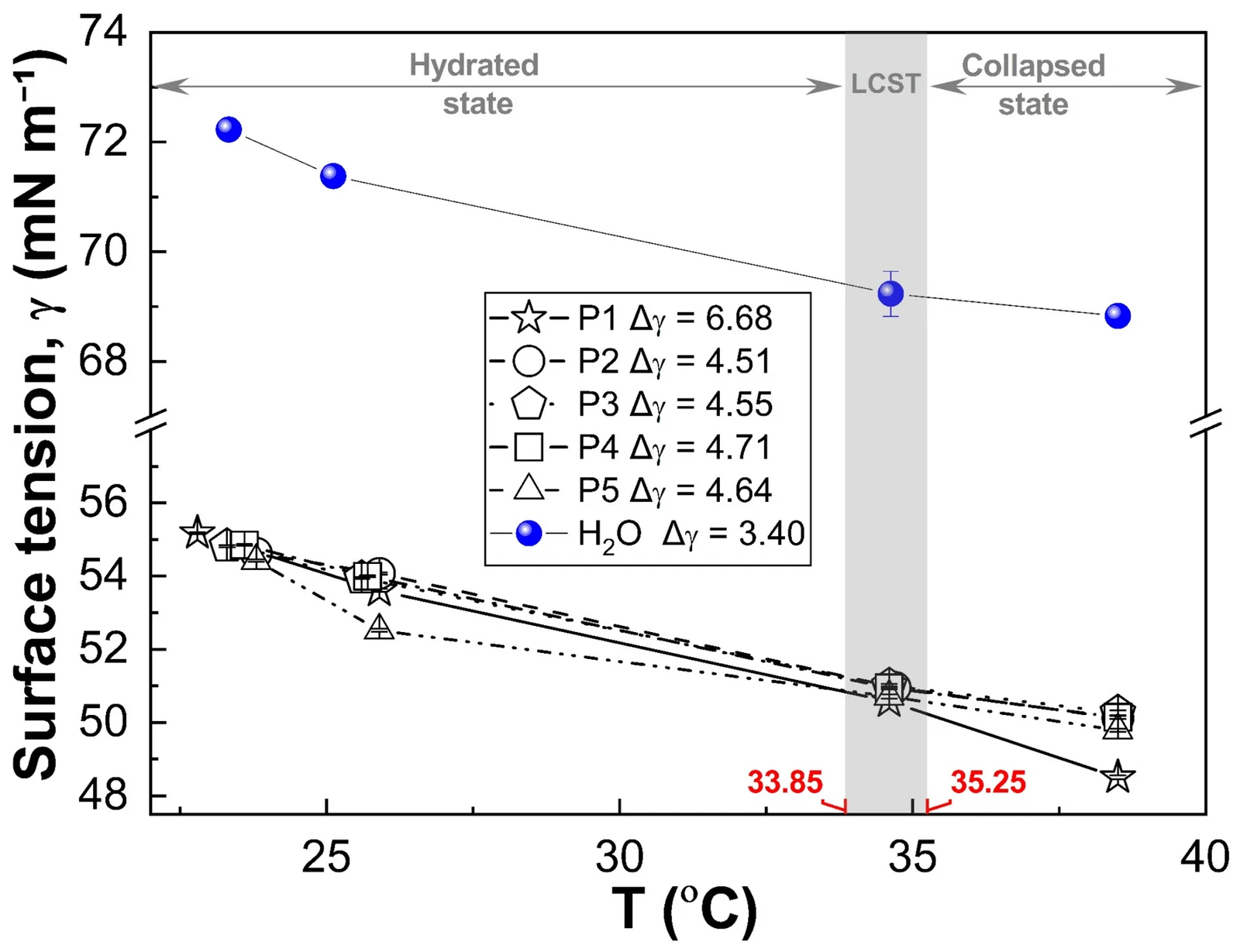
Temperature-dependent surface tension (γ) of polymer dispersions P1, P2, P3, P4, and P5 and deionized water used as a reference. Δγ represents the difference in surface tension between the lowest and highest measured temperatures.

Figure 8 shows the surface tension changes for all tested polymer dispersions, deionized water used as a reference at four temperatures, covering the range from approximately 22.8 to 38.5 °C, and the phase transition temperature region. The Δγ values correspond to the surface tension difference between the lowest and highest measured temperatures for each tested system. For comparison, the surface tension of deionized water decreased from approximately 72.2 to 68.8 mN m^−1^ over the investigated temperature range (Δγ = 3.40 mN m^−1^). All polymer dispersions exhibited markedly lower surface tension values than water, with Δγ ranging from 4.51 to 6.68 mN m^−1^.

### 3.7. RI Measurements

**Figure 9:**
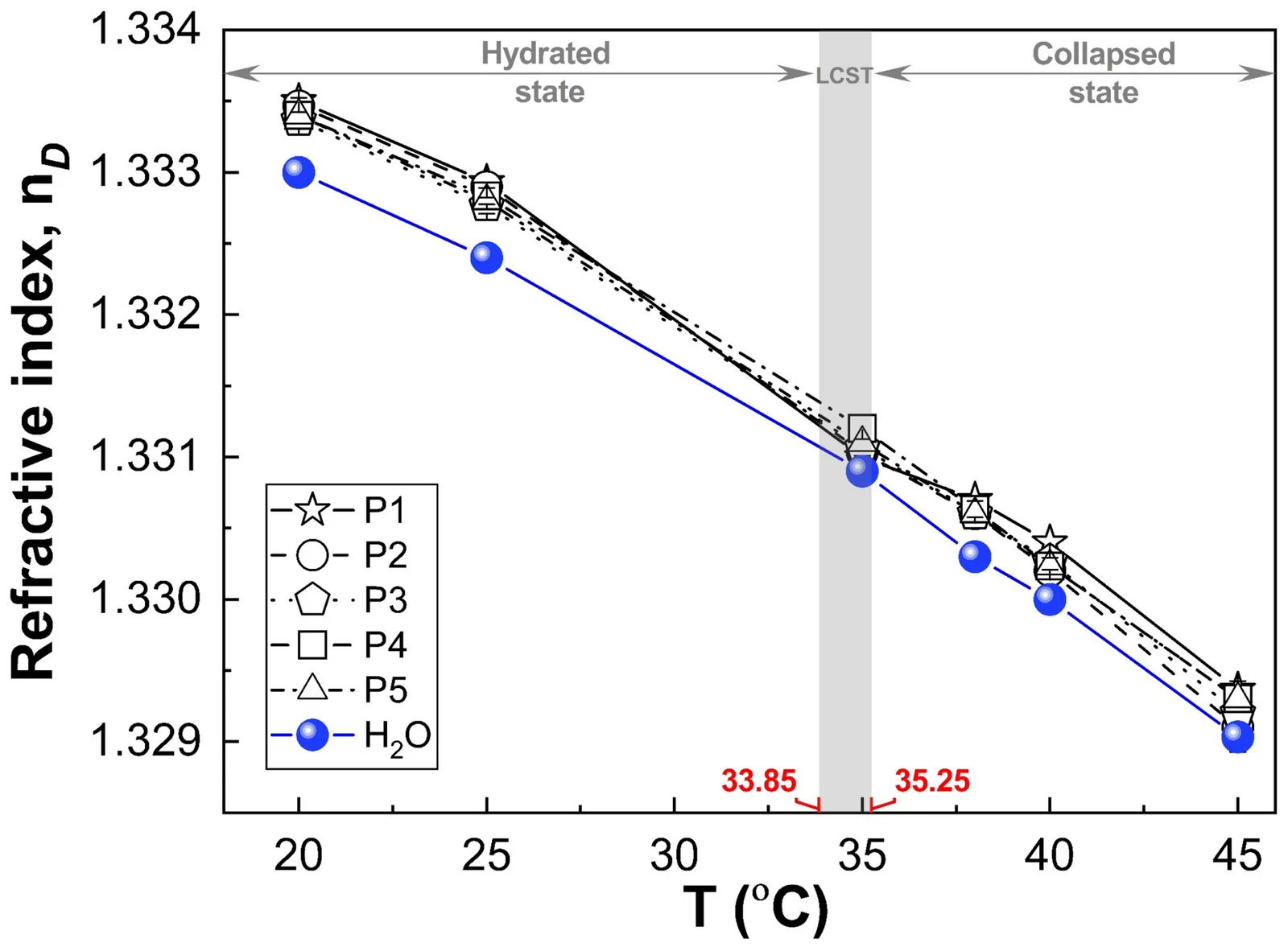
Changes in the refractive index (n_D_) with temperatures for P1, P2, P3, P4, and P5 polymeric samples and deionized water, as determined by digital refractometry.

The temperature-dependent changes in the refractive index of the P1–P5 polymer dispersions, in comparison with those of deionized water used as a reference, are presented in Figure 9.

### 3.8. ZP Measurements

**Figure 10:**
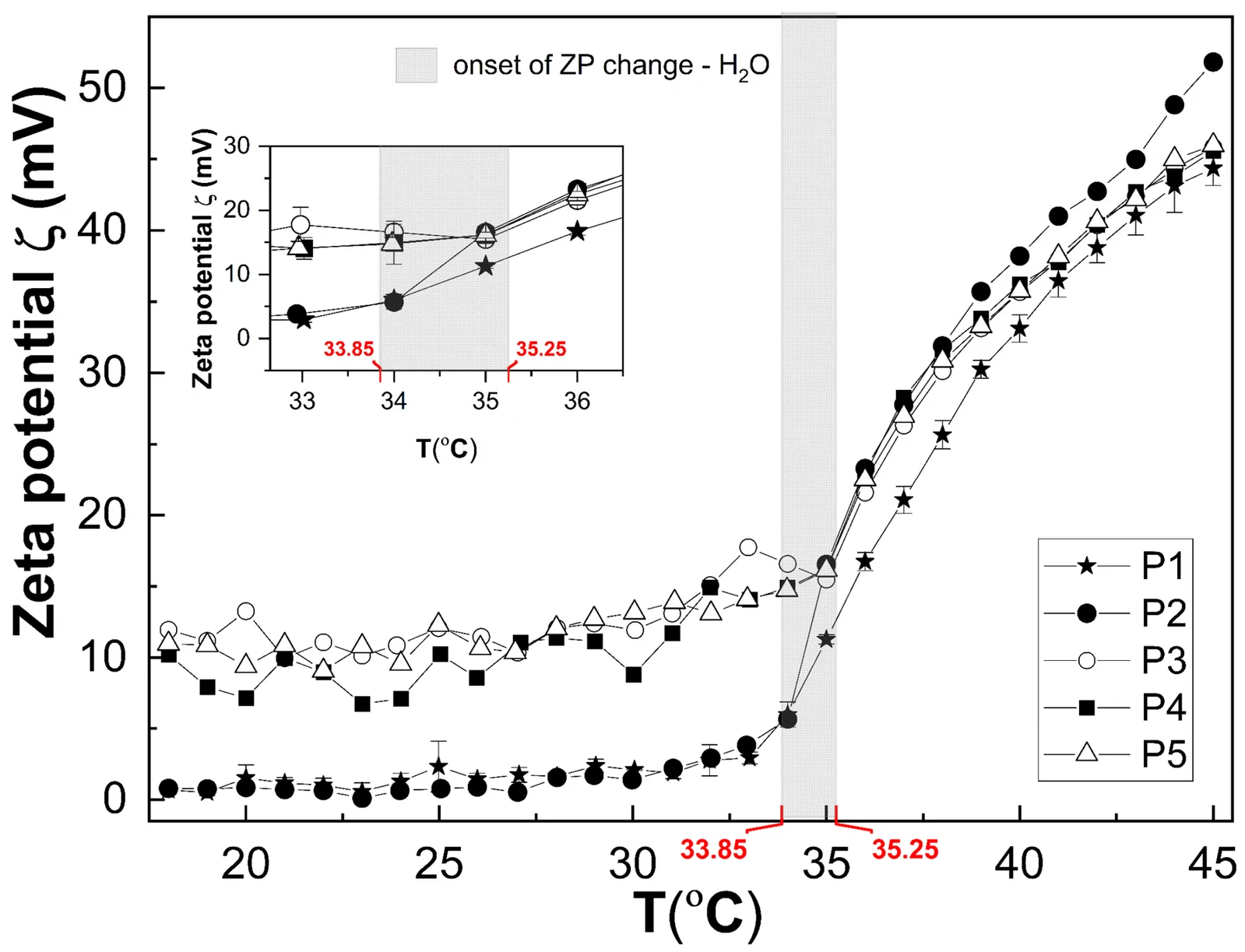
Alterations in zeta potential (ZP) in relation to temperatures of P1, P2, P3, P4 and P5 samples, as determined by electrophoretic mobility.

The effect of temperature on the zeta potential of the synthesized polymers is illustrated in Figure 10. It should be noted that the samples were not buffered during the measurement and that the pH values of the aqueous dispersions of P1, P2, P3, P4 and P5 were 6.50, 6.35, 6.71, 6.52, 6.71 respectively. Five measurements were taken for each temperature and the mean and standard error were calculated.

### 3.9. TEMs

**Figure 11:**
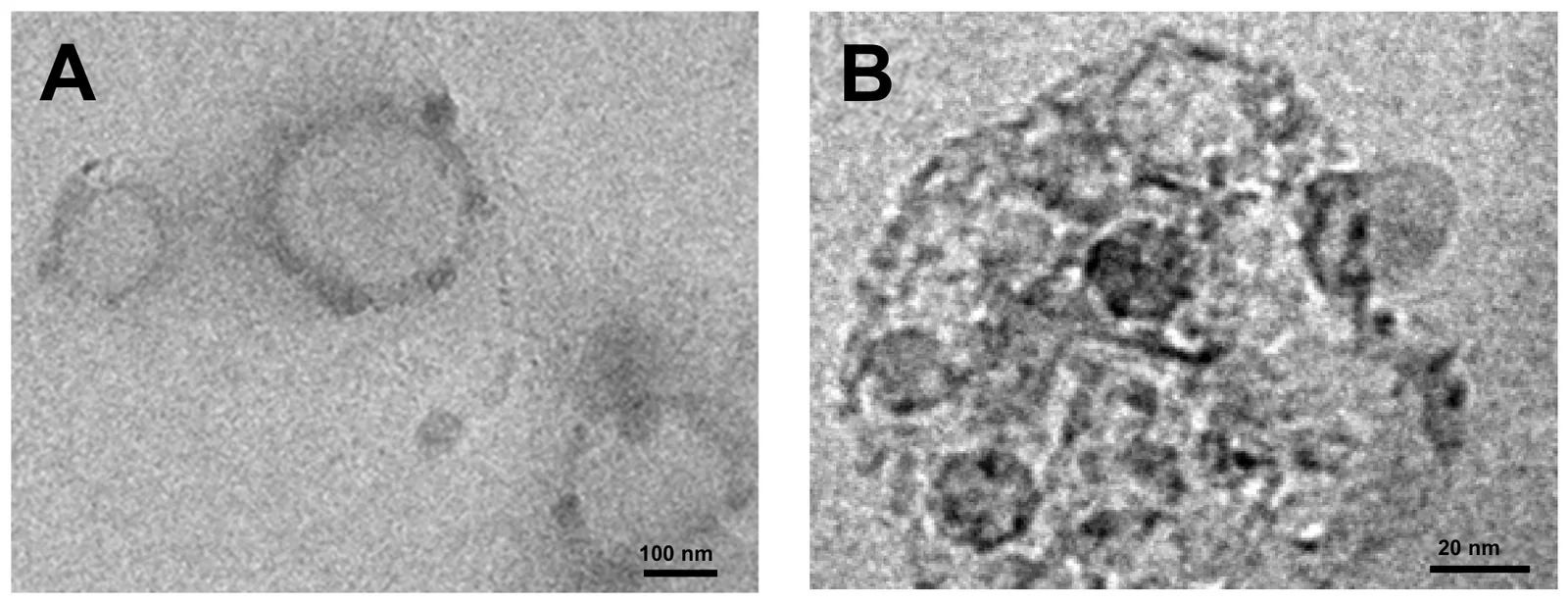

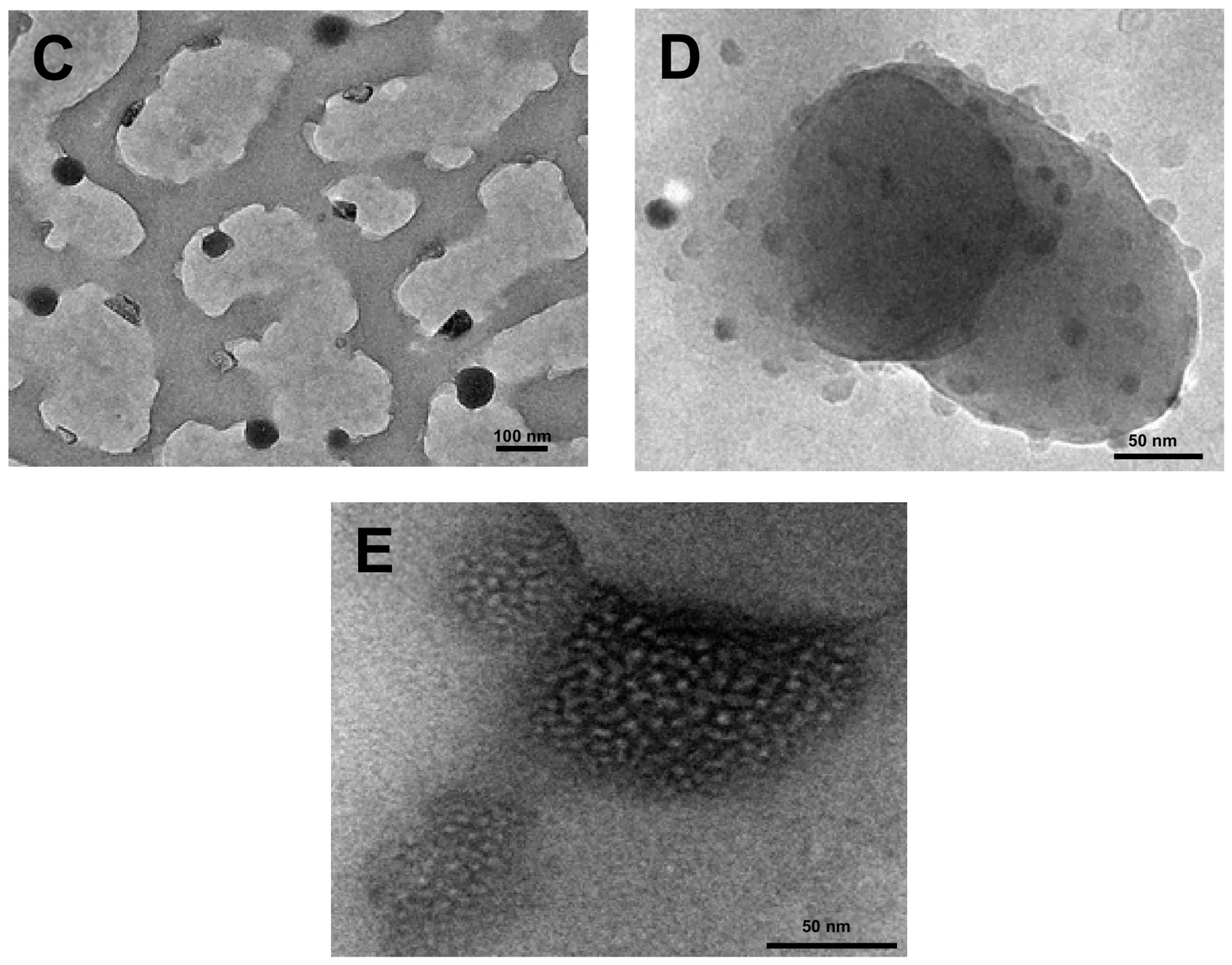
Representative TEM images of P1 (**A**), P2 (**B**), P3 (**C**), P4 (**D**) and P5 (**E**) polymers.

TEM was employed to examine the morphology of the obtained polymer particles. A typical TEM image can be seen in Figure 11.

### 3.10. HD and ZP Measurements in Different Dispersion Media

Temperature-dependent HD and ZP measurements of the synthesized polymers were additionally conducted in PBS and acetate buffer. The obtained results were then compared with those previously determined in water, thereby providing a direct comparison of the polymer behavior in different dispersion media.

**Figure 12:**
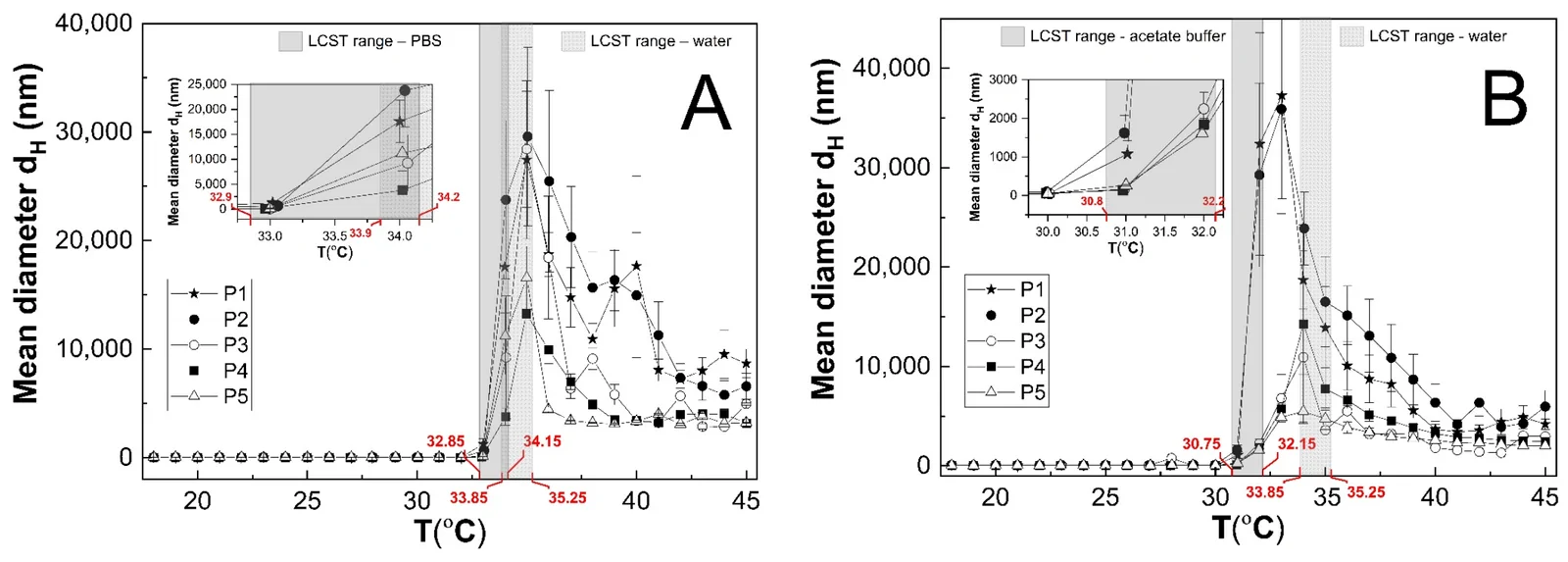
The alterations in hydrodynamic diameter (HD) in relation to temperatures of P1, P2, P3, P4, and P5 samples dispersed in (**A**) PBS and (**B**) acetate buffer, as determined by dynamic light scattering (DLS).

The temperature-dependent changes in HD of the polymer samples dispersed in PBS and acetate buffer are presented in Figure 12.

In PBS, an increase in HD was observed within the temperature range of approximately 32.85–34.15 °C, followed by pronounced particle aggregation, with maximum HD values reaching several tens of micrometers. In acetate buffer, the increase in HD occurred at lower temperatures, within approximately 30.75–32.15 °C, and was also accompanied by a marked increase in HD, consistent with extensive aggregation.

Compared with the transition range previously observed in water (33.85–35.25 °C), the corresponding changes in HD occurred at lower temperatures in both buffer media.

**Figure 13:**
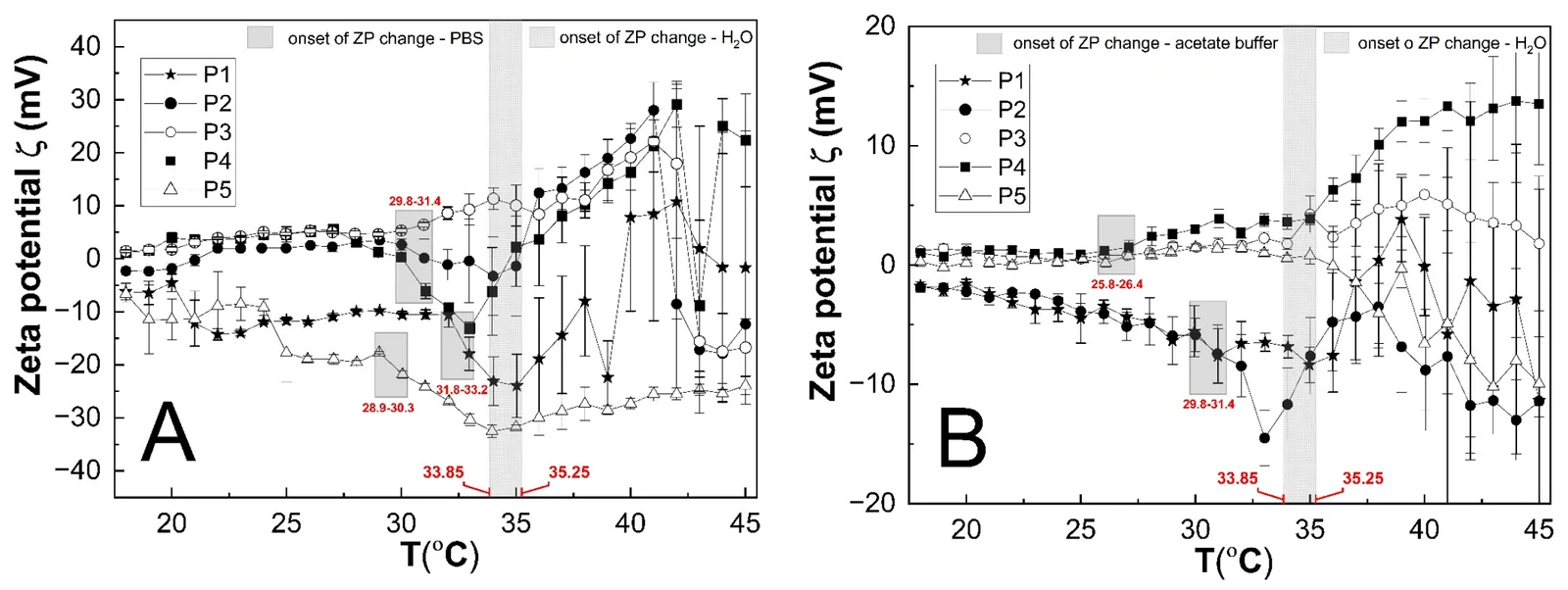
Alterations in zeta potential (ZP) in relation to temperatures of P1, P2, P3, P4, and P5 samples dispersed in (**A**) PBS and (**B**) acetate buffer, as determined by electrophoretic mobility (EM).

The temperature-dependent changes in ZP of the polymer samples dispersed in PBS and acetate buffer are presented in Figure 13.

In PBS and acetate buffer, the temperature-dependent changes in ZP exhibited greater heterogeneity among the investigated polymers than those previously observed in water. In the present study, the onset of ZP changes was observed at approximately 28.9–30.3 °C for P5, 29.8–31.4 °C for P2, P3, and P4, and 31.8–33.2 °C for P1. In acetate buffer, two distinct ranges were observed, with the onset of ZP changes occurring at approximately 25.8–26.4 °C for P3–P5 and at 29.8–31.4 °C for P1 and P2. In comparison, the onset of ZP changes in water was observed within a narrower and higher temperature range of 33.85–35.25 °C.

## 4. Discussion

### 4.1. Synthesis Analysis

The polymerization technique employed enables the synthesis of thermoresponsive polymer systems using a limited number of reagents [73]. From the outset, it was evident that thermosensitive polymers were obtained during the polymerization process. This was substantiated by the emergence of milky turbidity in the reaction mixture, which intensified over time, indicative of the propagation of polymer chains and the growth of polymeric particles. Moreover, it was observed that the opacity of the mixture disappeared when the temperature was reduced to below 35 °C, which is consistent with the predicted polymer properties characterized by LCST [74,75]. No linear relationship was identified between the time of turbidity appearance in the reaction mixture and the length of the cross-linker chain. Most researchers have used lower initiator concentrations, so the scope of observations for the interactions of small structures is limited, and they could be used as an interesting alternative to cast polymeric nanostructures or microstructures. The potential renal clearance of polymeric nanoparticles depends on several factors, including their hydrodynamic size and interactions with the biological environment [76,77].

The mass of solid products obtained by lyophilization from 100 mL of polymer dispersion ranged from 0.1294 g to 0.1567 g. The maximum mass of solid product was obtained from sample P1—0.1567 g, and the minimum was obtained from polymer P2—0.1294 g. It can be hypothesized that the most advantageous synthesis in terms of yield is the one with a cross-linker of mass Mn ~ 750.

### 4.2. Conductivity and Osmolality Analysis

The conductivity measurement, a method that was proven effective in our previous studies [72,78,79] was implemented to observe changes in conductivity during the polymerization process. However, it was subsequently observed that temperature-dependent conductivity profiles may also provide complementary information on changes occurring in the polymer system during cooling. As demonstrated in Figure 3, the distinct deflections observed in the conductivity profiles may indicate temperature-induced changes occurring within the polymer system. The investigation of the time–temperature dependence of conductivity during cooling of the post-reaction system to room temperature enabled the identification of characteristic changes in the conductivity profiles of each polymer system and determination of the time and temperature at which these changes occurred. Interestingly, distinct changes in the conductivity profiles during cooling were observed for the present NVCL-based systems synthesized using the cationic initiator AMPA. In our previous study of NVCL-based systems synthesized using the anionic initiator potassium persulfate (KPS) [80], no comparable changes in conductivity were observed during cooling under analogous measurement conditions. Similarly, no such alterations were recorded in our previous studies of NIPA-based systems [72,78,79]. The comparison with the previously investigated NVCL systems is particularly relevant, as it suggests that the occurrence of the characteristic conductivity changes cannot be attributed solely to the monomer type and may be influenced by the nature of the initiator. Nevertheless, the present results do not allow the specific mechanism underlying this effect to be established. The observed temperature-dependent conductivity changes may reflect alterations in the distribution and mobility of ionic species within the post-reaction system, potentially accompanied by temperature-induced structural reorganization of the polymer particles. However, even low concentrations of dissolved ionic species may substantially affect conductivity. The investigated post-reaction systems may contain products of polymerization side reactions as well as additional ionic species originating from the initiator, which should also be considered when interpreting the conductivity profiles.

In the course of the research conducted into changes in conductivity during the cooling process of the reaction mixture, two distinct changes were observed within the temperature range that was the subject of investigation. The presence of multiple changes in the conductivity profile may be indicative of successive structural or ionic rearrangements occurring during the cooling process. The thermal response of PNVCL-based systems is known to vary considerably with molecular weight, polymer composition and architecture, concentration, as well as the surrounding solution environment [81,82]. Consequently, the observed conductivity changes may reflect different stages of temperature-induced structural reorganization within the polymer system. However, conductivity measurements alone do not allow these changes to be unequivocally assigned to separate phase transitions.

All polymer dispersions following the reaction (P1–P5) exhibited a slightly acidic pH prior to the purification process. The aqueous solution of NVCL monomer tested prior to polymerization exhibited a pH of approximately 4.6. This observation indicates that during the synthesis process, not all monomer molecules polymerize, or alternatively, other ions or products are formed during radical reactions, slightly acidifying the reaction environment. Following dialysis purification, the average pH increased to 6.54, indicating a substantial reduction in the components responsible for the slightly acidic characteristic of the post-reaction system. These results support the effectiveness of the FED purification procedure following polymerization.

When evaluating the potential application of purified polymer dispersions in biological systems, their very low osmolality (2.0–2.6 mOsm kg^−1^) must also be taken into account, as the osmolality of the final preparation would require adjustment to the appropriate physiological range.

### 4.3. ATR-FTIR Analysis

As anticipated, the ATR-FTIR spectra of the synthesized P1–P5 polymers demonstrate a high degree of similarity. A comparison of the spectra obtained for the substrates and the resulting polymers reveals that the characteristic C=C absorption band of NVCL at 1652 cm^−1^ is absent in the polymer spectra. This observation is consistent with the consumption of vinyl double bonds during polymerization. Furthermore, the absorption bands originating from the vinyl group =CH and =CH_2_, located in the spectrum of the monomer at 3109 cm^−1^ and 993 cm^−1^, disappeared in the spectra of the polymers. The disappearance of these characteristic absorption bands provides further support for the occurrence of polymerization. The presence of the NVCL-derived structural units in the polymer chain is further supported by the presence in the polymer spectra of -CH_2_ peaks at 970 cm^−1^, indicating vibrations originating from the seven-membered caprolactam ring. The absorption bands observed at 1083 cm^−1^ and 1194 cm^−1^, attributed to C–O–C stretching vibrations, are consistent with the presence of PEGDMA-derived ether segments in the obtained polymer systems. Taken together, these spectral observations support polymer formation and are consistent with the presence of PEGDMA-derived segments, while quantitative assessment of conversion and effective crosslinking would require complementary analytical methods.

### 4.4. HD Analysis

**Figure 14:**
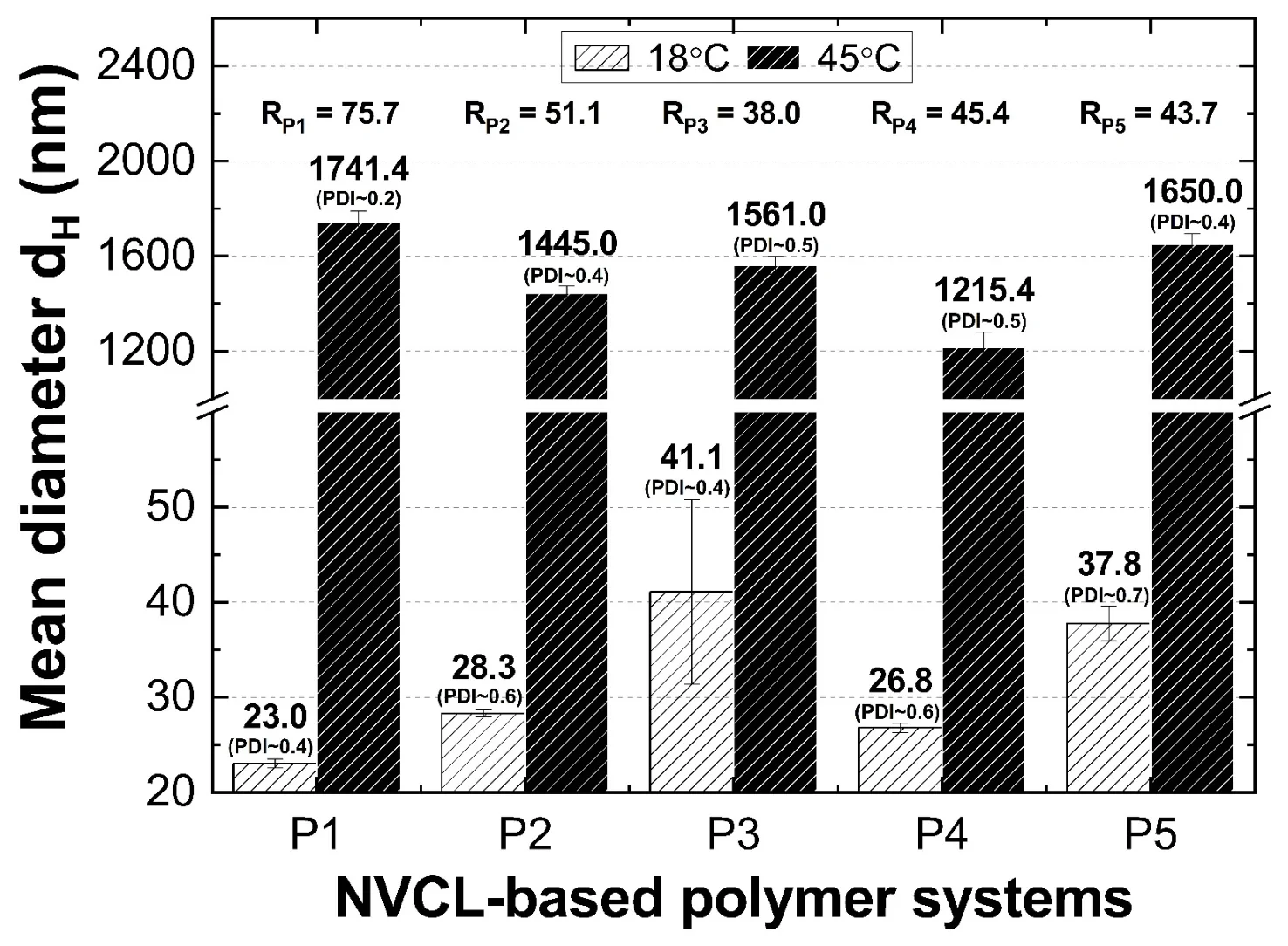
Hydrodynamic diameters (HDs) of P1–P5 at 18 °C and 45 °C (mean ± SD, n = 5), with corresponding PDI values and HD ratios (R = HD_45_/HD_18_). The nominal molecular weights (Mn) of PEGDMA used for P1–P5 were 750, 2000, 6000, 10,000, and 20,000, respectively.

The findings presented in this study offer substantiated evidence that the polymer particles obtained are of a nano-scale dimension (cf. Figure 5 and Figure 14). Furthermore, a phase transition temperature of between 34 and 35 °C has been determined.

The investigation revealed no discernible linear correlation between HD and the molecular weight of the cross-linker. It is noteworthy that a decrease in hydrodynamic diameter was observed in polymers P4 and P5, following a systematic increase in HD from P1 to P3. This non-monotonic behavior may be associated with differences in the spatial organization, chain mobility, and intermolecular interactions of the polymer systems prepared using PEGDMAs of different molecular weights. Furthermore, an increased intramolecular interaction may contribute to a decrease in effective hydration and hydrodynamic diameter [83]. In P4 and P5, the longer PEGDMA chains may provide greater conformational flexibility and promote intra- and intermolecular interactions, which could contribute to the observed differences in HD [84,85].

Differences in HD may also be influenced by kinetic factors operating during polymer formation. The rate of polymer chain termination can depend on the translational diffusion of macroradicals as well as on segmental motions of polymer chains and chain ends. Therefore, differences in PEGDMA molecular weight and chain mobility could affect these processes and, consequently, the dimensions of the resulting polymer particles. However, the present data do not allow the relative contribution of these mechanisms to the observed non-monotonic changes in HD to be determined.

The intensity-weighted hydrodynamic diameter distributions obtained by dynamic light scattering at 18 °C were broad and indicated the presence of at least two populations, comprising smaller dispersed particles and larger particles or agglomerates. Below the LCST, PNVCL chains are hydrated through interactions between their polar carbonyl groups and surrounding water molecules, including hydrogen bonding. As temperature increases, changes in the thermodynamic balance of polymer–water interactions make hydration progressively less favorable, promoting dehydration and conformational collapse of the PNVCL chains. This process may reduce the dimensions of individual primary polymer particles while increasing their tendency to associate through hydrophobic interactions. Consequently, the high HD values observed at elevated temperatures are likely to reflect the formation of aggregates or agglomerates of primary polymer particles rather than the dimensions of individual particles. The temperature-dependent hydrodynamic diameter distributions, including the narrowing observed at elevated temperatures, are consistent with this interpretation.

Although the ratio of aggregate to primary particle diameters may provide a simplified geometric estimate of the aggregation number, the intensity-weighted DLS distributions do not allow the actual number of primary particles within an aggregate to be determined. Such an estimate would require additional information on the dimensions of the collapsed primary particles and the internal structure of the aggregates.

The polymer particles contain both hydrophilic and hydrophobic groups and may therefore exhibit surface-active properties, and undergo self-organization in water through hydrophobic, polar and charge interactions. Such interactions may favor the formation of spherical micelle-like assemblies.

The coexistence of structures with different apparent dimensions, evident from the intensity-weighted HD distributions and TEM images presented in Figure 5B and Figure 11, may be consistent with particle growth or reorganization processes. One possible mechanism is Ostwald ripening, in which larger particles grow at the expense of smaller ones. However, the occurrence of this mechanism cannot be confirmed on the basis of the present experimental results.

### 4.5. Hysteresis Analysis

Thermal hysteresis measurements presented in Figure 7 were performed to evaluate the reversibility of temperature-induced structural changes in the investigated polymer systems. This parameter is particularly important when considering the potential application of these materials as thermoresponsive drug delivery systems or in other biomedical applications.

The phase transition temperatures determined from the inflection point parameter (x_0_) of the Boltzmann fitting curves were observed within a relatively limited temperature range (approximately 33–35.5 °C) for all investigated polymer systems. These values were consistent with the transition temperatures (approximately 34–35 °C) estimated from the initial increase in hydrodynamic diameter observed during non-hysteresis DLS measurements, where the onset of the phase transition was identified. The agreement between the onset temperature and the x_0_ values supports the consistency of the applied approach for characterizing the thermoresponsive behavior of the investigated polymers. The variation in PEGDMA molecular weight resulted in only moderate differences in the phase transition temperature. The small differences between the x_0_ values obtained during heating and cooling cycles were reflected in the low thermal hysteresis values, confirming the high degree of reversibility of the phase transition process.

The calculated hysteresis values (ΔT) were low for all investigated polymers, indicating that the thermally induced phase transition was highly reversible. The lowest hysteresis was observed for polymer P4 cross-linked with PEGDMA 10000 (ΔT = 0.03 °C), indicating an almost negligible difference between the transition temperatures determined during heating and cooling cycles. This suggests highly reversible structural changes and a rapid response of the polymer network to temperature variations. The remaining polymers exhibited slightly higher hysteresis values (ΔT = 0.76–1.79 °C); however, these values still indicate a high degree of reversibility of the temperature-induced transition.

All investigated polymers exhibited reversible temperature-dependent behavior within the studied temperature range. The observed differences in hysteresis may reflect variations in the structural relaxation of the polymer network during cooling, including delayed rehydration and reorganization of polymer chains after the phase transition.

No clear monotonic relationship was observed between PEGDMA’s molecular weight and hysteresis magnitude. Therefore, the observed differences are likely governed by a complex interplay between polymer chain mobility, network organization, and dehydration/rehydration processes rather than by the molecular weight of the crosslinking agent alone. During heating, dehydration of polymer chains exposes hydrophobic segments, promoting intermolecular interactions and aggregation. During cooling, water molecules gradually re-enter the polymer network; however, the disassembly of formed aggregates may proceed more slowly than their formation. The presence of PEGDMA provides a stable polymer network, while the restrictions imposed by crosslinking may influence chain rearrangement and delay the recovery of the initial hydrated state after cooling, contributing to the persistence of long-lived aggregated structures.

Interestingly, P5, which exhibited one of the highest thermal hysteresis values, also showed a broader and less pronounced conductivity minimum at the highest temperature (37.24 °C) among the investigated systems (see Figure 3). Although the conductivity measurements were performed in the post-reaction mixture and the hysteresis measurements were performed on purified polymer dispersions, they may both reflect slower structural reorganization during cooling in comparison with the remaining polymers.

Therefore, the observed hysteresis values should be interpreted as indicators of the reversibility and temperature-response characteristics of the polymer systems rather than as direct limitations of their potential application as drug carriers.

Comparison of the HD profiles obtained during hysteresis measurements with those recorded without a heating–cooling cycle (Figure 5A) revealed comparable HD values up to the phase transition temperature. However, above the LCST, substantially higher HD values were observed during hysteresis experiments. This difference suggests that the aggregation state of thermoresponsive polymers is strongly influenced by measurement conditions. The longer equilibration time and higher temperature resolution applied during hysteresis measurements may have provided sufficient time for the polymer systems to reach a different aggregation state or a quasi-equilibrium state. Prolonged exposure within the phase transition region may promote the formation and growth of larger aggregates.

Although the temperature hysteresis ranged from 0.03 to 1.79 °C for the investigated systems, the differences in aggregate size between the heating and cooling cycles were considerably larger. This suggests slow aggregate disassembly and/or the presence of long-lived metastable aggregated structures. These findings demonstrate that the reversibility of the transition temperature and the reversibility of the aggregation process represent related but distinct characteristics of thermoresponsive polymer systems.

### 4.6. ST Analysis

Surface tension measurements as a function of temperature showed that in all polymer systems studied, surface tension decreased with increasing temperature. For P2, P3, and P4, the decrease in surface tension with increasing temperature was approximately linear, while P1 and P5 exhibited some deviations from this trend. These deviations may reflect differences in the temperature-dependent reorganization of the polymer–water interface related to the network architecture.

Importantly, the surface tension of all polymer dispersions was markedly lower than that of deionized water throughout the investigated temperature range. Moreover, the decrease induced by heating was greater for the polymer dispersions (Δγ = 4.51–6.68 mN m^−1^) than for water (Δγ = 3.40 mN m^−1^), indicating that the observed changes cannot be attributed solely to the temperature dependence of the aqueous medium and may also reflect temperature-induced changes in polymer hydration and organization.

Despite differences in PEGDMA molecular weight, surface tension values remained very similar near the phase transition temperature (34.6–34.7 °C), ranging from 50.58 to 51.00 mN m^−1^ for P1–P5. This indicates that PEGDMA molecular weight did not markedly affect the interfacial properties of the polymer dispersions within the phase transition temperature range.

However, some differences were observed between the polymer systems at the remaining measurement temperatures. At a temperature of approximately 25.9 °C, P5, cross-linked with PEGDMA 20000, exhibited a lower surface tension (52.52 mN m^−1^) than the other systems, for which values ranged from 53.61 to 54.07 mN m^−1^. At 38.5 °C, P1, cross-linked with the shortest PEGDMA (750 Da), exhibited the lowest surface tension (48.50 mN m^−1^), while the other polymers exhibited values ranging from 49.79 to 50.27 mN m^−1^. Although these differences were relatively small, they may suggest that the molecular weight of PEGDMA influences the organization of the polymer network at the polymer–water interface. This effect may be related not only to the molecular weight of the cross-linker but also to the resulting network architecture, chain mobility, and the accessibility of hydrophobic polymer segments at the interface.

The observed differences in surface tension were substantially smaller than the differences observed in hydrodynamic diameter and thermal hysteresis. For example, P1 and P4, which differed markedly in thermal hysteresis (1.79 and 0.03 °C, respectively), exhibited very similar surface tension values at approximately 34.6 °C (50.58 and 50.94 mN m^−1^, respectively). This indicates that hysteresis behavior is likely more strongly related to the kinetics of structural rearrangement, aggregation, and subsequent rehydration of the polymer network than to changes in interfacial properties.

Overall, the results suggest that variations in PEGDMA molecular weight may influence the thermoresponsive behavior of the polymer networks while their overall macroscopic interfacial properties remain relatively comparable near the phase transition temperature.

### 4.7. RI Analysis

The refractive index of all investigated polymer dispersions decreased progressively with increasing temperature, following the general trend observed for deionized water. No pronounced discontinuity in the refractive index profiles was observed within the phase transition region. However, at approximately 35 °C, the refractive index values of the polymer dispersions approached those measured for water, resulting in a minimum difference between the apparent refractive index of the dispersions and that of the aqueous reference.

The observed convergence occurred within the temperature range in which the investigated PNVCL-based systems underwent their thermoresponsive transition. In aqueous PNVCL systems, this transition is associated with changes in polymer hydration, conformational collapse, and subsequent aggregation. Therefore, the refractive index behavior observed in the present study may reflect changes in the optical properties of the dispersions accompanying the temperature-induced reorganization of the polymer network. However, refractive index measurements alone do not allow the underlying changes in polymer hydration or phase composition to be quantified directly. The refractive index measurements therefore provide complementary information on the temperature-dependent optical properties of the investigated polymer dispersions.

### 4.8. ZP Analysis

This study demonstrated that the charge on the surface of the polymer particles remained positive over the entire studied temperature range of 18–45 °C. The positive charge is likely associated with cationic groups derived from the water-soluble cationic azo initiator AMPA used during polymer synthesis. As the investigated polymers form spherical particles, the positively charged groups of the initiator may be distributed not only on the surface of the particles, but may also be located inside the particles in the spaces of the polymer network formed by the cross-linkers. According to the recorded data, the zeta potential values during heating cycles may reflect the LCST range; however, the critical temperatures were assessed from HD measurements.

A clear temperature-dependent increase in zeta potential was observed, particularly above the phase transition temperature (cf. Figure 10). Upon exceeding the LCST, the polymer network undergoes conformational collapse and dehydration, potentially altering the spatial distribution and accessibility of charged groups within the spherical particles. Furthermore, at elevated temperatures, the hydrogen bonds that may shield charges on the surface of the particles are broken, resulting in a diminished shielding effect. The consequence of this process is an accumulation of charge at the surface, resulting in an increase in charge density and, by extension, an escalation of the aggregation process. This phenomenon aligns with the main principles of DLVO theory, which assumes that the total interaction energy between colloidal particles is the sum of van der Waals (attractive) and electrical double-layer (repulsive) interactions [86]. The resulting energy barrier is therefore proportional to the size of the particles. Consequently, smaller particles have a lower aggregation barrier, i.e., they are more susceptible to aggregation [87]. This assertion is corroborated by temperature-dependent HD studies (cf. Figure 5). A temperature-dependent increase in HD above the phase transition temperature was observed, which is related to aggregation and the detection of large structures such as agglomerates rather than small particles in DLS studies. In addition, a temperature-dependent increase in ZP values was observed. At 45 °C, the absolute ZP values reached 44–52 mV; however, these values alone do not establish colloidal stability, particularly in view of the extensive aggregation detected by DLS. This assertion is corroborated by zeta potential measurements at temperatures above the phase transition temperature, where an increase in zeta potential is observed, reaching absolute values of 44 to 52 mV at 45 °C. These values indicate that the polymer dispersion exhibits good stability [88,89,90]. As can be seen, modification of the charge on the surface of a particle has the potential to affect its physicochemical properties, including loss of repulsion forces and an increase in aggregation. This modification may also have consequences for the particle’s behavior when it comes into contact with living cells. The utilization of positively charged drug carriers has been demonstrated to exhibit enhanced stability in the presence of biological cations, as well as augmented interaction with negatively charged biological membranes [91,92,93]. This, in turn, has been shown to result in increased drug transport capacity [94,95]. However, it should be noted that a positive charge may also have deleterious effects, as intensive interaction with a biological membrane can lead to membrane damage and toxicity [96,97]. Recent findings have indicated that the toxicity of particles is not solely determined by their positive zeta potential value, but rather by the charge density at the particle’s surface [98]. This suggests that the cationic charge alone is insufficient to induce toxicity. Consequently, further investigation into synthesized polymeric particles, with a focus on charge density and toxicity, is imperative.

At 18 °C, polymer molecules with lower-molecular-weight PEGDMA (Mn ~ 750 and 2000) exhibited ZP values close to zero. In contrast, polymers cross-linked with higher-molecular-weight PEGDMA showed ZP values of approximately 10 mV. This effect may be due to the influence of the cross-linker chain length on network flexibility. These differences may also be associated with variations in the polymer network architecture and network flexibility, which could influence the accessibility of charged groups and their interactions with ions present in the surrounding medium.

### 4.9. TEM Analysis

The TEM images of the P1–P5 polymer samples (Figure 11A–E) revealed predominantly rounded features with heterogeneous apparent dimensions, including smaller structures associated with larger assemblies. These observations provide qualitative information on the morphological appearance of the investigated polymer systems.

An interesting feature is the association of smaller structures with larger ones, resulting in morphological patterns resembling hierarchical particle assemblies described in the literature [99,100]. Such similarities may suggest possible hierarchical organization of the polymer structures; however, this interpretation remains hypothetical and cannot be confirmed solely on the basis of conventional TEM observations.

The observed morphological heterogeneity may be associated with processes occurring during polymer particle formation and growth. Local variations in polymer concentration and polymer–water interactions during precipitation polymerization could promote the association of growing polymer chains and the formation of polymer-rich domains. Such processes might contribute to differences in the morphology of the resulting structures. Previous studies have described the synthesis of thermoresponsive microgels [101] and morphological variations in related polymer systems prepared using different cross-linkers [102]. However, these possible mechanisms remain hypothetical and cannot be established from the present TEM observations.

Since the TEM specimens were prepared by depositing aqueous polymer dispersions onto carbon-coated copper grids followed by air drying, possible drying-induced collapse, deformation, aggregation, and redistribution of the polymer material cannot be excluded. Consequently, the observed morphological features should be interpreted as qualitative characteristics of the dried samples rather than direct evidence of specific particle architectures or hierarchical organization in aqueous dispersion.

### 4.10. HD and ZP Analysis in Different Dispersion Media

The dispersion medium exerted a pronounced influence on both the thermoresponsive and electrokinetic behavior of the polymer systems under investigation. In comparison with water, in which the HD-derived transition range was observed at 33.85–35.25 °C, the corresponding changes occurred at lower temperatures in PBS (32.85–34.15 °C) and acetate buffer (30.75–32.15 °C). In addition to this shift, both buffer media promoted substantially stronger aggregation above the transition region than that observed in water. These findings indicate that the thermoresponsive behavior of the investigated polymers is strongly dependent on the surrounding aqueous environment rather than being determined solely by the polymer network structure.

The influence of the dispersion medium was also evident in the temperature-dependent ZP profiles. In water, the onset of ZP changes occurred within a relatively narrow temperature range that closely corresponded to the HD-derived transition region. Conversely, the electrokinetic response in phosphate-buffered saline (PBS) and acetate buffer exhibited greater heterogeneity and was found to be contingent upon the polymer system. In particular, within the context of the acetate buffer, alterations in ZP for certain polymers were discernible at temperatures that fell well below the transition range ascertained from HD. This observation suggests that alterations in the electrokinetic properties of the particles may precede the pronounced changes in hydrodynamic diameter associated with the thermoresponsive transition and subsequent aggregation.

The observed changes in the thermoresponsive behavior of the investigated polymers in the buffer media may be associated with changes in polymer–water interactions induced by the ionic environment. The phase transition of PNVCL is closely related to changes in polymer hydration and the balance between hydrophilic and hydrophobic interactions. The presence and type of dissolved ions can substantially modify this balance [81]. As demonstrated in preceding studies, the presence of salts has been shown to substantially affect the solvation of PNVCL. Salts can promote phase separation at lower temperatures, consequently resulting in a decrease in the transition temperature [81]. In particular, PNVCL has been reported to exhibit a lower transition temperature in PBS than in water, while studies performed in citrate, acetate, and phosphate buffers demonstrated that the ionic environment may exert a stronger influence on the transition than pH alone [81]. This finding is consistent with the present results, in which the HD-derived transition range shifted from 33.85–35.25 °C in water to 32.85–34.15 °C in PBS and 30.75–32.15 °C in acetate buffer. Consequently, the disparities observed between PBS and the acetate buffer should not be ascribed exclusively to pH, but rather to the cumulative effects of medium composition, ionic environment, and polymer–solvent interactions.

The dispersion medium also had a pronounced effect on the extent of particle aggregation above the transition region. While the maximum HD values in water were generally in the micrometer range, substantially larger structures, reaching several tens of micrometers, were detected in the buffer media. Such behavior may be indicative of diminished colloidal stability in the presence of elevated ionic conditions, a phenomenon that may be compounded by temperature-induced dehydration and the subsequent collapse of the PNVCL-based network. As demonstrated in preceding studies on PNVCL systems, primary globular structures formed above the transition temperature have been observed to undergo further aggregation into larger agglomerates. Furthermore, the presence of salts has been shown to promote the aggregation of PNVCL nanogels above their volume phase transition temperature [103]. Consequently, the substantial HD values evident in PBS and acetate buffer are indicative of the formation of higher-order aggregates, as opposed to merely reflecting the dimensions of individual collapsed polymer particles.

From an application perspective, the effects of the dispersion medium on the transition behavior, aggregation, and electrokinetic properties of the investigated polymers are particularly relevant for their potential use as drug delivery carriers. Depending on the intended route of administration, such systems may be exposed to environments with markedly different pH, ionic composition, and ionic strength, including those encountered at the skin surface, vaginal mucosa, and within physiological fluids. The observed shifts in the HD-derived transition range and differences in aggregation and ZP indicate that the behavior determined in purified water alone may not fully reflect that expected under application-relevant conditions. Further studies involving media representative of the intended site of administration, drug loading and release characteristics, and biological compatibility are necessary to evaluate the suitability of these polymer systems for specific pharmaceutical applications.

## 5. Conclusions

As a consequence of SFPP, five thermosensitive NVCL-based polymer systems (P1–P5) were obtained using PEGDMAs of different molecular weights (Mn 750–20,000) as a crosslinking agent. ATR-FTIR analysis revealed spectral changes consistent with polymerization, including the disappearance of characteristic vinyl absorption bands, although the degree of conversion and effective crosslinking could not be determined quantitatively by this method. Conductivity measurements during cooling of the post-reaction system revealed characteristic temperature-dependent changes, providing complementary information on their thermal response. DLS and electrophoretic mobility measurements performed at 18–45 °C demonstrated pronounced temperature-dependent changes in HD, PDI, and ZP, with the main thermoresponsive transition occurring at approximately 33–35 °C in water. The aqueous polymer dispersions exhibited positive ZP values (0.11–51.81 mV), while the mean HD below the transition region ranged from 21.40 to 40.48 nm. Thermal hysteresis measurements demonstrated reversible thermoresponsive behavior with low hysteresis values (ΔT = 0.03–1.79 °C).

Surface tension decreased with increasing temperature for all investigated systems, while the values remained very similar among P1–P5 near the phase transition temperature. Refractive index measurements likewise showed a temperature-dependent decrease in RI, with only small differences among the investigated polymers and relative to water.

The dispersion medium markedly affected polymer response, shifting the HD-derived transition range to lower temperatures in PBS (32.85–34.15 °C) and the acetate buffer (30.75–32.15 °C) and promoting substantially stronger aggregation than in water. The electrokinetic response was also more heterogeneous in the buffer media, demonstrating the importance of the surrounding aqueous environment for the behavior of these polymer systems.

Overall, variations in PEGDMA molecular weights were associated with differences in the physicochemical properties of the investigated polymer systems; however, no simple monotonic relationship between PEGDMA molecular weight and the measured parameters was observed. The results also demonstrate the pronounced influence of the surrounding aqueous environment on the thermoresponsive behavior of these materials. Since the internal network architecture and effective crosslinking density were not quantitatively determined, their relationship with the observed physicochemical properties remains to be established.

The purified polymer dispersions exhibited pH values of 6.35–6.71 and very low osmolality (2.0–2.6 mOsm kg^−1^); therefore, adjustment of osmolality would be required when developing formulations for biomedical applications.

## Figures and Tables

**Table 1:** Substrate compositions of P1, P2, P3, P4, and P5 cross-linked polymers.

Components		Type of Co-Polymer Nanoparticle System				
		P1	P2	P3	P4	P5
Monomer (g)	NVCL	3.0007	3.0003	3.0004	3.0003	3.0030
Cationic initiator (g)	AMPA	1.4606	1.4613	1.4603	1.4612	1.4613
Cross-linkers (g)	PEGDMA (Mn ~ 750)	0.0081	-	-	-	-
	PEGDMA (Mn ~ 2000)	-	0.0216	-	-	-
	PEGDMA (Mn ~ 6000)	-	-	0.0647	-	-
	PEGDMA (Mn ~ 10,000)	-	-		0.1078	-
	PEGDMA (Mn ~ 20,000)					0.2155

**Table 2:** Inflection point temperatures (x_0_) obtained from Boltzmann fitting and thermal hysteresis values for NVCL-based polymers.

Type of Polymer Nanoparticle System	x_0_ Heating (°C)	x_0_ Cooling (°C)	ΔT Thermal Hysteresis (°C)	R^2^ Heating	R^2^ Cooling
P1	33.6895 ± 0.0042	35.4763 ± 0.0214	1.7868	0.9999	0.9999
P2	33.7194 ± 0.0089	32.9552 ± 0.0030 *	0.7642	0.9998	0.9999
P3	34.1487 ± 0.0409	35.0221 ± 0.1780	0.8734	0.9871	0.9685
P4	34.5252 ± 0.0674	34.5575 ± 0.0187	0.0323	0.9999	0.9998
P5	34.2850 ± 0.0128	35.4460 ± 0.1873	1.1610	0.9999	0.9987

* dx constrained during fitting.

## Data Availability

The original contributions presented in this study are included in the article. Further inquiries can be directed to the corresponding author.

## Abbreviations

AMPA	2,2′-azobis[2-methylpropionamidine] dihydrochloride
ATR-FTIR	attenuated total reflectance Fourier-transform infrared spectroscopy
DLaTGS	deuterated L-alanine-doped triglycine sulfate detector
DLS	dynamic light scattering
DLVO	Derjaguin–Landau–Verwey–Overbeek
EM	electrophoretic mobility
FED	forced equilibrium dialysis
HD	hydrodynamic diameter
KPS	potassium persulfate
LCST	lower critical solution temperature
LDV	laser Doppler velocimetry
Mn	number-average molecular weight
MWCO	molecular weight cut-off
NIBS	non-invasive back scatter
NIPA	*N*-isopropylacrylamide
NVCL	*N*-vinylcaprolactam
PBS	phosphate-buffered saline
PDI	polydispersity index
PEG	poly(ethylene glycol)
PEGDMA	poly(ethylene glycol) dimethacrylate
PNVCL	poly(*N*-vinylcaprolactam)
RI	refractive index
SD	standard deviation
SFPP	surfactant-free precipitation polymerization
ST	surface tension
STEM	scanning transmission electron microscopy
TEM	transmission electron microscopy
UCST	upper critical solution temperature
ZP	zeta potential
